# Performance Optimization Analysis of Partial Discharge Detection Manipulator Based on STPSO-BP and CM-SA Algorithms

**DOI:** 10.3390/s25165214

**Published:** 2025-08-21

**Authors:** Lisha Luo, Junjie Huang, Yuyuan Chen, Yujing Zhao, Jufang Hu, Chunru Xiong

**Affiliations:** 1 School of Mechanical and Energy Engineering, Guangdong Ocean University, Yangjiang 529500, China; 43821223zz@stu.gdou.edu.cn (L.L.); 43821409uu@stu.gdou.edu.cn (J.H.); 2 School of Computer Science and Engineering, Guangdong Ocean University, Yangjiang 529500, China; 44021105ff1@stu.gdou.edu.cn (Y.C.); yujing.zhao0823@gmail.com (Y.Z.); xcrmcu@gdou.edu.cn (C.X.)

**Keywords:** partial discharge detection, manipulator inverse kinematics, STPSO-BP neural network, end-positioning error compensation, energy consumption optimization

## Abstract

In high-voltage switchgear, partial discharge (PD) detection using six-degree-of-freedom (6-DOF) manipulators presents challenges. However, these involve inverse kinematics (IK) solution redundancy and the lack of synergistic optimization between end-effector positioning accuracy and energy consumption. To address these issues, a dual-layer adaptive optimization model integrating multiple algorithms is proposed. In the first layer, a spatio-temporal correlation particle memory-based particle swarm optimization BP neural network (STPSO-BP) is employed. It replaces traditional IK, while long short-term memory (LSTM) predicts particle movement trends, and trajectory similarity penalties constrain search trajectories. Thereby, positioning accuracy and adaptability are enhanced. In the second layer, a chaotic mapping-based simulated annealing (CM-SA) algorithm is utilized. Chaotic joint angle constraints, dynamic weight adjustment, and dynamic temperature regulation are incorporated. This approach achieves collaborative optimization of energy consumption and positioning error, utilizing cubic spline interpolation to smooth the joint trajectory. Specifically, the positioning error decreases by 68.9% compared with the traditional BP neural network algorithm. Energy consumption is reduced by 60.18% in contrast to the pre-optimization state. Overall, the model achieves significant optimization. An innovative solution for synergistic accuracy–energy control in 6-DOF manipulators for PD detection is offered.

## 1. Introduction

High-voltage switchgear, a critical component of power systems, often exhibits partial discharge (PD) phenomena during the accident latency period [1]. Current detection technologies for PD in high-voltage switchgear primarily include transient earth voltage (TEV), acoustic emission (AE), and ultra-high-frequency (UHF) detection [2]. However, traditional PD detection methods for high-voltage switchgear rely on manual inspection, which faces issues such as low efficiency, insufficient accuracy, and safety hazards. Consequently, manipulators with advanced PD detection sensors have emerged as an effective alternative for monitoring high-voltage switchgear systems. Such manipulators can collect internal data of high-voltage switchgear in real-time, analyze insulation conditions, achieve high-precision positioning, flexibly adapt to complex environments, reduce manual operation demands, and ensure the safe operation of power equipment [3]. However, for six-degree-of-freedom (6-DOF) manipulators used in PD detection, the redundant inverse kinematics (IK) solution poses significant challenges to the precise positioning of the end-effector.

Traditional analytical methods face practical limitations such as cumbersome, redundant solution derivation and poor adaptability to dynamic tasks. Scholars in China and elsewhere have researched the IK of redundant manipulators, proposing various methods to address redundancy in manipulators’ IK. Chen et al. [4] proposed a unified, systematic kinematic modeling method for a novel 6R manipulator. This method separates couples joint variables without increasing the degree; however, it requires meeting specific degree reduction conditions, which limit its applicability to a certain extent. Additionally, the BP neural network algorithm has unique advantages in solving the IK of redundant manipulators owing to its excellent nonlinear fitting capability. Gao et al. [5] proposed an IK solution based on the BP neural network. By comparing the prediction results of different training algorithms, the limitation of traditional IK algorithms in achieving a unique optimal solution was successfully circumvented. To address the issue that the conventional gradient descent method in BP neural networks quickly falls into local optimality, numerous scholars have attempted to combine it with intelligent optimization algorithms. Bai et al. [6] proposed an algorithm for solving IK based on the FOA-optimized BP neural network, effectively overcoming shortcomings of traditional BP neural networks, such as the tendency to fall into local minima during IK solving, with the final output error being smaller than that of conventional BP neural network algorithms. Lin et al. [7] proposed an inverse solution algorithm based on an adaptive spider wasp optimization (ASWO)-optimized backpropagation (BP) neural network, effectively addressing the issue of BP neural networks falling into local optimality and significantly improving positioning accuracy. However, due to the inherent limitations of optimization algorithms, when faced with complex manipulator workspaces and multiple constraints, the algorithms are prone to falling into local optimality, resulting in solutions that are not globally optimal.

Ensuring detection precision and long-term endurance is paramount when manipulators perform PD detection tasks. Therefore, energy consumption optimization planning strategies are critical, as they can effectively reduce energy consumption and enhance endurance. Although relevant studies have explored energy consumption optimization, none have fully addressed the core requirement of precision. Wang et al. [8] proposed an energy optimization method based on parallel deep reinforcement learning (DRL), achieving a 23.21% reduction in energy consumption. However, its end-positioning accuracy was not considered, and the transfer accuracy between simulated and real scenarios remains unvalidated, making it unsuitable for the strict precision demands of detection tasks. Li et al. [9] framed industrial robot trajectory planning as a nonlinear optimization problem under dynamic constraints, solved via the sequential quadratic programming (SQP) method. This approach reduced energy consumption by 13.95% while improving trajectory smoothness. However, it focuses solely on energy consumption as the optimization objective, neglects positioning error control, and is time-intensive, limiting its adaptability to dynamically changing detection scenarios. Xu et al. [10] developed a time-energy dual-objective planning method for freight train cleaning robots, combining seventh-order polynomial interpolation with the improved Harris hawk optimization (IHHO) algorithm. Optimization efficiency was boosted through an adaptive jump strength attenuation strategy. However, prioritizing operational efficiency led to an 18.59% increase in energy consumption. Additionally, its weak generalization to complex paths fails to meet the core demand for low energy consumption in detection tasks.

This paper proposes a dual-layer adaptive optimization model integrating multiple algorithms to address critical issues in redundant IK of 6-DOF robotic arms. These issues include the tendency of intelligent optimization algorithms to be trapped in local optima and the challenge of synergistically optimizing end-positioning accuracy and energy consumption. The first layer constructs an end-positioning error compensation model: a particle swarm optimization with a spatiotemporal particle memory mechanism (STPSO) optimizes a backpropagation (BP) neural network to replace traditional IK calculations. A long short-term memory (LSTM)-based velocity prediction model dynamically infers particle trends from historical optimal positions to overcome traditional PSO’s local search limits. At the same time, a trajectory similarity penalty term suppresses convergence and enhances global exploration, optimizing BP’s weights and biases for higher accuracy. The STPSO-BP neural network is used to learn pose–joint angle mappings via training, avoiding analytical solutions and simplifying redundant problem handling. The second layer uses a chaos-mapped simulated annealing (CM-SA) algorithm to optimize energy consumption and positioning error based on the first layer’s optimal joint angles. Chaotic-mapping dynamic weight adjustment and dual-objective temperature regulation ensure solution feasibility and balance positioning error and energy consumption. Finally, cubic spline interpolation smooths optimized trajectories for motion continuity.

This paper is organized as follows. Section 2 introduces the detection framework of the 6-DOF PD detection manipulator. Section 3 presents its forward kinematics model. Section 4 details end-effector positioning error compensation for the manipulator using the STPSO-BP algorithm. Section 5 establishes the manipulator’s energy consumption optimization model. Section 6 proposes the dual-layer adaptive optimization model integrating positioning error compensation and energy consumption optimization. Section 7 reports experimental results of the dual-layer model in actual PD detection of high-voltage switchgears by the manipulator. Section 8 presents the results and discussion. Finally, Section 9 concludes the paper.

## 2. 6-DOF PD Detection Manipulator’s Detection Work Framework

The working system of the 6-DOF PD detection manipulator employs a hierarchical structure divided into the application and data layers.

**Figure 1:**
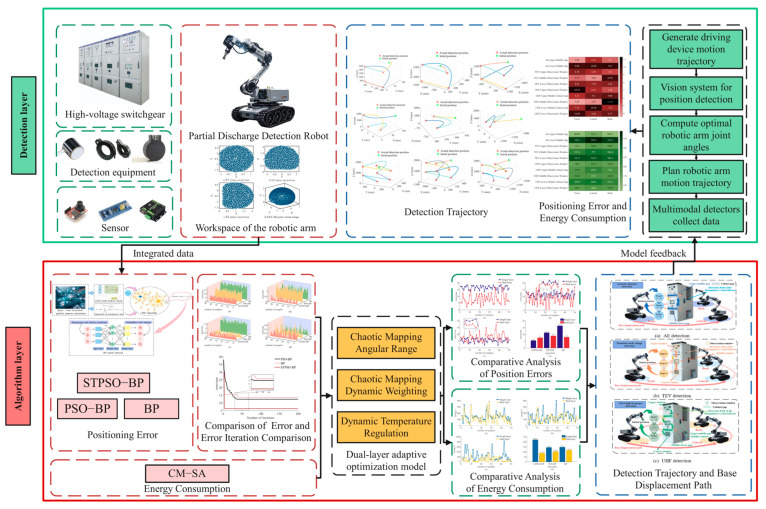
The working framework of the 6-DOF PD detection robot.

As shown in Figure 1, the application layer interacts with the physical environment of the high-voltage switchgear via terminal devices to execute detection tasks. First, the global motion trajectory of the manipulator driving device is pre-generated based on the 3D position information of the high-voltage switchgear. Detection points are then identified by the vision module, with the target coordinates derived from the 3D data.

The data layer provides algorithm support and applies a two-layer adaptive optimization model to optimize the terminal positioning error and energy consumption, achieving a dynamic balance. First, the Latin Hypercube model determines the reachable range of the manipulator to verify the accessibility of the target. Then, the target coordinates are input into the model and solved using the STPSO-BP and CM-SA algorithms to obtain the optimal joint angles, which guide the manipulator operations. Specifically, STPSO-BP first generates high-precision initial joint angles through IK. CM-SA then optimizes energy consumption within the accuracy constraints set by STPSO-BP, with mutual feedback between them, iteratively refining the optimal joint angles.

Upon reaching the targets, AE, TEV, and UHF detections are performed using broadband differential, TEV, and KPD2-UHF sensors. The data are transmitted in real-time during acquisition. When the detection data meet the preset confidence thresholds, the manipulator switches targets until all are completed and then returns to its initial pose to organize the data. Finally, the end-effector’s trajectory, positioning error, and energy consumption are visually presented.

## 3. Analysis and Determination of the Working Range of a 6-DOF PD Detection Manipulator

### 3.1. Specification Parameters of the 6-DOF PD Detection Manipulator

**Figure 2:**
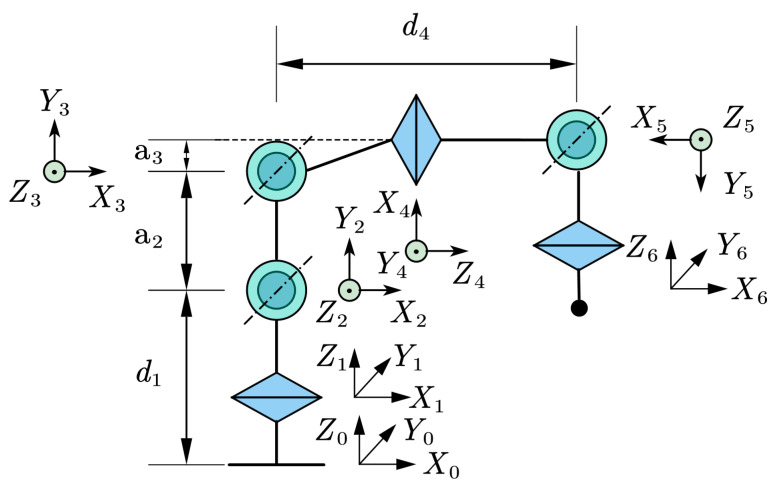
Links in the coordinate system of the manipulators.

The PD detection manipulator is a 6-DOF serial manipulator consisting of a waist, shoulder, elbow, forearm, wrist, and wrist rotation joints. To address the specific requirements of high-voltage switchgear inspection, a tracked chassis was integrated to enhance mobility, with the offset of joint 1 (*d*_1_) adjusted to 89.5 cm. The base link was extended to increase the vertical working range, accommodating large vertical spans in detection areas. The Denavit–Hartenberg (D-H) method was employed to establish the coordinate system, as illustrated in Figure 2, with parameters detailed in Table 1. Based on the manipulator’s structural characteristics and the joint angle limitations, the range of values for each joint variable is shown in Table 2. The unloaded manipulator weighs 35 kg and features a replaceable end-effector for various detection methods. Key components of the electronic control system are specified in Table 3.

As shown in the tables below, the following four parameters define the coordinate system of each link: (1) link length a, that is, the length of each common perpendicular; (2) link twist angle *a*, that is, the included angle between adjacent *Z*-axis.; (3) link offset d, that is, the distance between adjacent common perpendiculars; and (4) joint angle θ, that is, the rotation angle around the *Z*-axis.

Information regarding the manufacturers of key components in the robotic arm’s electronic control system is as follows: The RE50, RE40, EC-i52, and EC-i40 motor models, the MR-L encoder, GP planetary reducers (including versions with worm and worm gear assemblies as well as standard versions), and the Epos2 50/5 driver are supplied by Maxon Motor (Sachseln, Switzerland). The FHA-8C motor model, the incremental photoelectric encoder integrated into the FHA-8C motor, and harmonic reducers are provided by Harmonic Drive Systems (Tokyo, Japan). The HEDS5540 encoder is manufactured by Broadcom Inc. (Palo Alto, CA, USA). The ACJ-55-18 driver is supplied by Copley Controls (Boston, MA, USA). Multi-axis motion control cards are provided by Delta Tau (Los Angeles, CA, USA).

### 3.2. Establishment of the Forward Kinematic Model

In the kinematic research of manipulators, forward kinematics modeling is crucial for determining the position and orientation of the end-effector in the base coordinate system [11]. To achieve forward kinematics modeling, this paper adopts the Denavit–Hartenberg (D-H) parameter method. The general expression of the link transformation matrix is shown in Equation (1).

(1) $$A_{i}=\left[\begin{array}{cccc} \cos\theta_{i} & -\sin\theta_{i}\cos a_{i} & \sin\theta_{i}\sin a_{i} & a_{i}\cos\theta_{i}\\ \sin\theta_{i} & \cos\theta_{i}\cos a_{i} & -\cos\theta_{i}\sin a_{i} & a_{i}\sin\theta_{i}\\ 0 & \sin a_{i} & \cos a_{i} & d_{i}\\ 0 & 0 & 0 & 1 \end{array}\right]$$

For a 6-DOF serial manipulator, the D-H matrices corresponding to each link are denoted as *A*_1_, *A*_2_, *A*_3_, *A*_4_, *A*_5_, and *A*_6_. By the principle of kinematic recursion, the pose transformation of the manipulator end relative to the base coordinate system can be realized by successively left-multiplying the adjacent link transformation matrices. Ultimately, the homogeneous transformation matrix T60 from the base coordinate system to the end-effector coordinate system is obtained, and its expression is shown in Equation (2) [12].

(2) T  60=T  10T  21T  32T  43T  54T  65=∏i=16Ai=nxoxaxpxnyoyaypynzozazpz0001

Among them, ${n=[n_{x},n_{y},n_{z}]}^{T},{o=[o_{x},o_{y},o_{z}]}^{T}$ and ${\;a=[a_{x},a_{y},a_{z}]}^{T}$ represent the direction vectors of the end-effector along the *X*-axis, *Y*-axis, and *Z*-axis, respectively, in the base coordinate system. ${\;p=[p_{x},p_{y},p_{z}]}^{T}$ represents the position coordinates of the end-effector in the base coordinate system. Through the process mentioned above, the forward kinematics modeling of the robotic arm can be completed. Specifically, when the rotation angles of each joint of the robotic arm are given, the position and orientation of the end-effector in the base coordinate system can be determined.

### 3.3. Determination of the Working Range of a Robotic Arm Based on Latin Hypercube

A 6-DOF PD detection manipulator model was built using MATLAB 2022b with the Robotic Toolbox. Based on the above forward kinematics model, this paper uses Latin Hypercube Sampling (LHS) to randomly sample each joint rotation range, verifying the manipulator’s workspace coverage. LHS is a uniform sampling method for high-dimensional spaces. It divides each dimension into equal intervals and randomly selects a point in each interval to ensure sample uniformity. Compared to traditional random sampling, LHS can enhance sample diversity, reduce training redundancy, and facilitate the BP neural network algorithm’s faster and more stable convergence [13].

According to the rotation intervals of each manipulator joint in Table 2, the manipulator’s workspace was derived using LHS. Based on the random number rule, the joint angle generation formula is $\theta =\theta +(\theta_{\max}-\theta_{\min})Rand(N,1)+\theta_{\min}$, yielding a specific number of random joint angle values. Subsequently, the end position and posture of the manipulators were further calculated via manipulator forward kinematics, and the points corresponding to these end poses were depicted in space to determine the working space of the robot arm.

**Figure 3:**
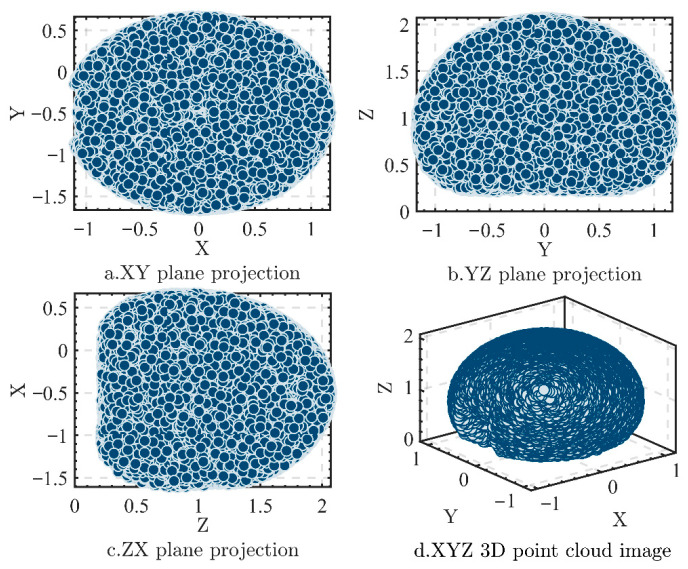
Workspace view of the manipulators. (**a**) XY plane projection; (**b**) YZ plane projection; (**c**) ZX plane projection; (**d**) XYZ 3D point cloud image.

Figure 3a presents a 3D point cloud formed by randomly selecting N = 10,000 reachable points within the joint rotation ranges using LHS. The workspace of the manipulators for PD detection was approximately spherical. Figure 3b–d show that the projections on the three planes exhibit a relatively uniform distribution without obvious defects. From Figure 3, the working range of this detection manipulator is roughly *x* ∈ [−1107, 1167] mm, *y* ∈ [−1169, 1175] mm, *z* ∈ [213, 2070] mm. By moderately lengthening the base link, the workspace of the manipulators in the vertical direction is significantly extended.

This paper selects the familiar KYN28A-12 switchgear (650 × 1500 × 2300 mm). Vertically, joint 1 (d_1_ = 89.5 cm), joint 4 (d_4_ = 47.7 cm), and joint 2 (−150°–15°) enable end-effector coverage from *z* = 213 mm to 2070 mm. This height range covers over 90% of the switchgear’s total height, meeting detection needs for its components from bottom to top. Horizontally, the main link a_2_ = 65.8 cm is key for the manipulator’s forward extension. The extension ranges of joint 2 (−150°–15°) and joint 5 (−120°–120°) enhance horizontal swing, enabling scanning of the whole 650 mm wide surface. Short link a_3_ = 3.5 cm improves compact folding in confined spaces, aiding access to observation windows or narrow gaps. Moreover, 360° continuous rotation of joint 6 enables precise end-effector alignment in any posture.

In summary, the structural parameters and joint motion ensure complete coverage of the switchgear’s front surface in height and width. Combined with reachable workspace simulation in Figure 3, this structure meets spatial accessibility requirements for PD detection, verifying feasibility and adaptability in confined spaces.

## 4. Compensation Analysis of End-Effector Positioning Error for PD Detection Manipulators Based on the STPSO-BP Algorithm

**Figure 4:**
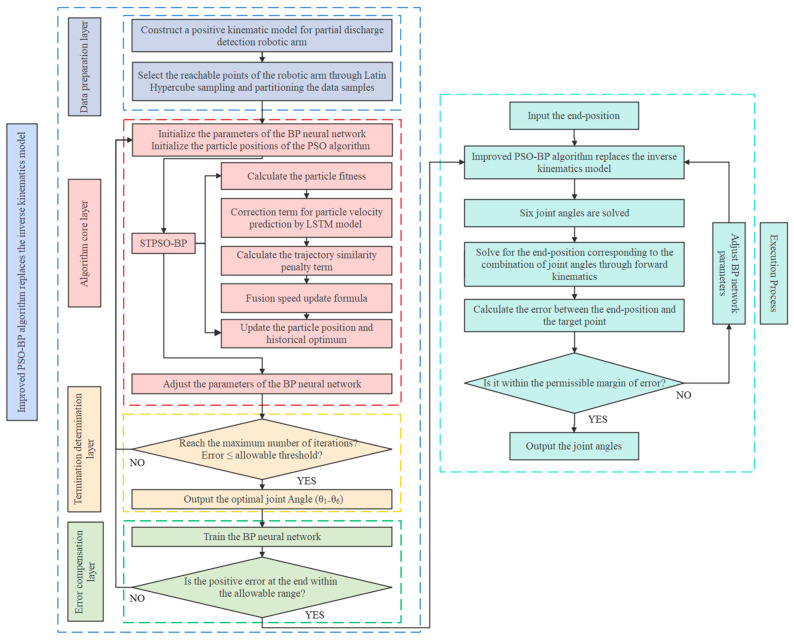
End-effector error compensation model based on STPSO-BP algorithm.

In PD testing of high-voltage switchgears, various detection methods demand high-precision positioning of the manipulator’s end-effector [14]. In TEV detection, sensor displacement can cause errors in TEV signal acquisition. In AE detection, acoustic wave signals may be missed if the sensor deviates from the cabinet gaps or observation windows. Similarly, in UHF detection, sensor deviation from the observation window can lead to the loss of key electromagnetic wave signals, affecting the accurate assessment of the switchgear’s operating conditions. Consequently, this paper proposes an IK model based on the STPSO-BP algorithm for the end-effector error compensation of a PD detection manipulator. This model was developed according to detection methods and a manipulator structure. Based on the end-effector position coordinates and posture information, it solves in reverse for the six joint angles that minimize end-effector positioning errors, thereby replacing the traditional IK approach. The modeling process is shown in Figure 4.

### 4.1. IK Solution Based on BP Algorithm

The IK of manipulators belongs to a multi-input, multi-output, nonlinear complex system [15]. Traditional IK algorithms have limited practicality and are computationally intensive [16]. However, the BP neural network possesses strong nonlinear fitting and fault-tolerant capabilities, making it suitable for solving complex, nonlinear, strongly coupled IK problems [17]. Thus, this paper substitutes a BP neural network for traditional IK algorithms, leveraging BP’s nonlinear fitting ability. It maps end-effector poses [*x*, *y*, *z*] to joint angles via data-driven learning, bypassing complex symbolic derivations and inherently addressing redundancy.

**Figure 5:**
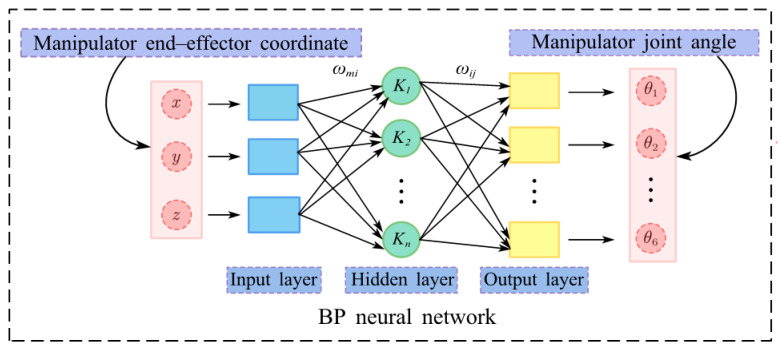
Schematic diagram of BP neural network structure.

In the structure of this neural network, neurons in the hidden layer adopt the Sigmoid function as the activation function, whereas the transfer function of the output layer is the Tansig function. After the spatial pose [*x*, *y*, *z*] of the end-effector of the PD detection manipulators is trained via the BP neural network, the six joint angles $\left[\theta_{1},\theta_{2},\theta_{3},\theta_{4},\theta_{5},\theta_{6}\right]$ of the manipulators for PD detection are obtained. A schematic of the network structure is presented in Figure 5, where the hidden layer contains seven neurons, the output layer contains six neurons, $\omega_{mi}$ represents the weight between the input and hidden layers, and $\omega_{ij}$ denotes the weight between the hidden and output layers. Details of the training dataset are provided in Section 4.5.

### 4.2. IK Solution Based on PSO-BP Algorithm

To address the issues that the BP neural network algorithm is susceptible to, including initial weights and biases, as well as local optima, this paper proposes a hybrid algorithm (PSO-BP) that utilizes the PSO algorithm for optimizing the BP neural network [18]. Within the PSO framework, particle parameters comprise the BP neural network’s weight matrices and bias vectors, with the particle position vector denoted as *x_i_*. The PSO algorithm employs the Mean Squared Error (MSE) of the robotic arm’s IK problem as the fitness function, which is defined as the global discrepancy between the end-effector coordinates obtained from the joint angles corresponding to the pose input and the actual target coordinates.

Through the collaborative mechanism of the PSO algorithm, particles track both their historical optimal position and the global historical optimal position *gbest*, dynamically updating their motion states. This process balances global exploration and local exploitation in the solution space. The velocity update of particles is determined jointly by the inertia term, individual cognitive term, and social cognitive term, as shown in Equation (3). The new position of each particle is generated by adjusting the current velocity. Equation (4) describes the positional movement process of particles in the solution space, which iteratively approaches the optimal parameter combination step by step.

(3) $$v_{i}\;(t+1)=w\cdot v_{i}\;(t)+c_{1}r_{1}\;(pbest_{i}-x_{i}(t))+c_{2}r_{2}(gbest-x_{i}(t))$$

(4) $$x_{i}(t+1)=x_{i}(t)+v_{i}(t+1)$$

where $v_{i}\left(t\right)$ is the velocity vector of the i-th particle at the iteration number t, which controls the search direction and step-size. $c_{1}$ and $c_{2}$ are learning factors.$r_{1}$ and $r_{2}$ are random numbers following the uniform distribution $U\left(0,1\right)$. $x_{i}\left(t\right)$ is the position vector of the i-th particle at the iteration number t. $v_{i}\left(t+1\right)$ is the updated velocity of the i-th particle. $x_{i}\left(t+1\right)$ is the position vector of the i-th particle at the iteration number t+1. w is the inertia weight, and its value decays linearly with the iteration number, as shown in Equation (5).

(5) $$w=w_{\max}-\frac{(w_{\max}-w_{\min})\cdot t}{T_{\max}}$$

where $w_{max}$ and $w_{min}$ are the maximum and minimum inertia weights, respectively. t is the current iteration number, and $T_{max}$ is the maximum iteration number.

### 4.3. IK Solution Based on STPSO-BP Algorithm

**Figure 6:**
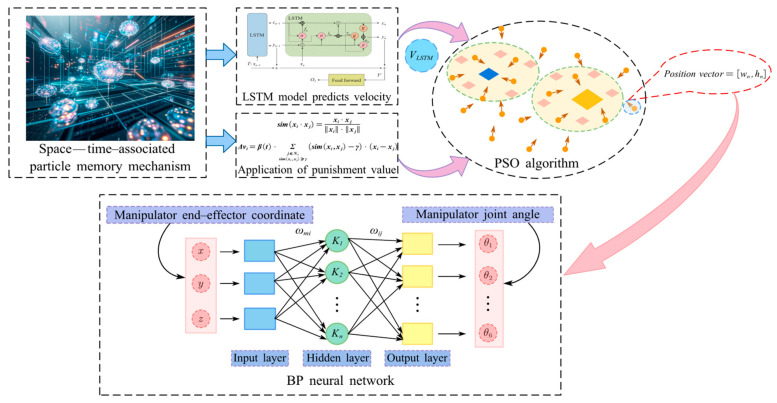
STPSO-BP algorithm for IK solution.

The PSO algorithm has demonstrated excellent performance in optimizing the parameters of BP neural networks. However, particle updates primarily rely on current information, neglecting historical search experiences. This oversight may lead to repeated ineffective searches or erroneous explorations [19]. Additionally, the PSO algorithm tends to become trapped in local optimal solutions when addressing complex multimodal functions or the IK problems of robotic arms with multiple local optima [20]. To address these issues, this paper proposes a spatio-temporal correlated particle memory mechanism to enhance the PSO-BP algorithm. An LSTM model is introduced in the temporal dimension to leverage historical information and improve the memory ability of the search process. Simultaneously, a trajectory similarity penalty term is added in the spatial dimension to suppress particle similarity and reduce ineffective searches. The IK solution process based on the STPSO-BP algorithm is illustrated in Figure 6.

#### 4.3.1. Historical Optimal Trajectory Matrix of Particles

The historical optimal trajectory of each particle records the evolution process of the particle’s position within the time step, and it forms a matrix, as shown in Equation (6). To adapt to the sequential processing characteristics of the LSTM model, the two-dimensional historical trajectory matrix needs to be converted into a three-dimensional tensor. Specifically, the trajectory matrix $HERT_{i}\;\in {\mathbb{R}}^{D\;\times \;T}$ of each particle is transposed, resulting in an input structure $HERT_{i}^{T}\in {\mathbb{R}}^{T\;\times \;D}$ with the time step as the first dimension, as shown in Equation (7).

(6) $$HERT_{i}=\left[\begin{array}{cccc} p_{1,1} & p_{1,2} & \ldots & p_{1,T}\\ p_{2,1} & p_{2,2} & \ldots & p_{2,T}\\ \vdots & \vdots & \ddots & \vdots \\ p_{D,1} & p_{D,2} & \ldots & p_{D,T} \end{array}\right]\in {\mathbb{R}}^{D\;\times \;T}$$

(7) $$X_{train}=\left[HERT_{1}^{T},HERT_{2}^{T},\ldots ,HERT_{N}^{T}\right]\in {\mathbb{R}}^{N\;\times \;T\;\times \;D}$$

where D is the dimension of particle parameters, T is the time step, and $p_{d,t}$ represents the optimal value of the d-th parameter at the t-th time step, and N is the size of the particle swarm.

#### 4.3.2. Individual Velocity Prediction Model Based on LSTM

LSTM captures temporal dependencies of parameter evolution, mapping historical trajectories to speed adjustments [21]. In architecture, LSTM comprises three layers: input, LSTM hidden, and fully connected output layers. The input layer has one layer with five neurons. The LSTM hidden layer (1 layer, 50 neurons) excels in temporal modeling, learning long-term dependencies in particle trajectories. The output layer is fully connected (1 layer, 5 neurons), ensuring complete prediction of current velocity updates. To boost training efficiency and convergence, LSTM uses a learning rate of 0.01, batch size 10, max iterations 1000, Levenberg–Marquardt (trainlm) optimizer, and target error 10^−6^. In training, the velocity prediction model minimizes MSE between *v*_LSTM_ and *v*_true_; Equation (8) shows the loss function.

(8) $$L=\frac{1}{N}\sum_{i=1}^{N}{\left\|v_{LSTM}^{\left(i\right)}-v_{ture}^{\left(i\right)}\right\|}^{2}$$

where $v_{LSTM}^{\left(i\right)}$ is the predicted velocity vector of the *i*-th particle by LSTM; $v_{ture}^{\left(i\right)}$ is the historical velocity vector of the *i*-th particle; and $∥\cdot {∥}^{2}$ is the L2-norm, which calculates the Euclidean distance between vectors.

In the velocity prediction stage, for the current particle *i*, the three-dimensional tensor $X_{train}$ is input into the LSTM model. Subsequently, the LSTM model outputs the predicted velocity $v_{LSTM}$. Finally, this predicted velocity $v_{LSTM}$ is incorporated as an adaptive term into the PSO velocity update formula, along with a linear decay strategy, as shown in Equations (9) and (10).

(9) $$\alpha =\alpha_{\max}-\frac{(\alpha_{\max}-\alpha_{\min})\cdot t}{T_{\max}}$$

(10) $$v_{i}(t+1)=w\cdot v_{i}(t)+c_{1}r_{1}(pbest_{i}-x_{i}(t))+c_{2}r_{2}(gbest-x_{i}(t))+\alpha \cdot v_{LSTM}$$

where $\alpha_{max}$ and $\alpha_{min}$ are the maximum and minimum velocity weights, respectively, T is the current iteration number, and $T_{max}$ is the maximum iteration number.

#### 4.3.3. Trajectory Similarity Penalty Term

In the STPSO-BP algorithm, particles integrate the sequential prediction capability of LSTM with the swarm search mechanism of PSO, significantly enhancing global exploration efficiency. However, when particle trajectories become highly convergent, swarm diversity decreases, potentially causing the algorithm to converge to local optima [22]. To address this, a trajectory similarity penalty term is proposed in this paper. By dynamically suppressing excessive inter-particle similarity, particles are compelled to explore different regions of the solution space, thereby balancing the diversity and convergence efficiency of the search process.

In this paper, cosine similarity is adopted to quantify the consistency of trajectory directions among particles [23]. For particles i and j, the similarity of their current position vectors $x_{i},x_{j}\in {\mathbb{R}}^{D}$ is defined as shown in Equation (11).

(11) $$sim(x_{i},x_{j})=\frac{x_{i}\cdot x_{j}}{\left\|x_{i}\right\|\cdot \left\|x_{j}\right\|}$$

where sim(⋅)∈[−1,1]; the larger the value, the more the movement directions of particles tend to converge.

The set of neighboring particles ${\mathbb{N}}_{i}$ consists of the k particles (excluding particle i itself) that are closest to particle i in terms of Euclidean distance. If the similarity between particle *i* and a neighboring particle $j\in N_{i}$ exceeds the similarity threshold, and the swarm diversity significantly decreases, a repulsive force in the opposite direction is applied to enhance particle diversity. The specific formula is shown in Equation (12).

(12) $$\Delta v_{i}=\beta (t)\cdot \sum_{\begin{array}{c} j\in N_{i}\\ sim(x_{i},x_{j})\geq \gamma \end{array}}\left(sim(x_{i},x_{j})-\gamma \right)\cdot (x_{i}-x_{j})$$

where k is the neighborhood scale parameter and is set as 5. γ is the similarity threshold and is set as 0.9. $\left(x_{i}-x_{j}\right)$ is the direction of repulsion. $\beta \left(t\right)$ is the dynamic penalty coefficient, adjusted according to a piecewise linear strategy. In the initial exploration stage, a high penalty coefficient drives particles to explore the solution space extensively. In the later exploitation stage, a reduced penalty coefficient allows particles to focus on local refined search, as shown in Equation (13).

(13) $$\beta (t)=\left\{\begin{array}{l} 0.5\;\;\;\;\;\;\;\;\;\;\;\;(t<0.3T_{\max})\\ 0.5-0.4\cdot \frac{t-0.3T_{\max}}{0.4T_{\max}}\;\;\;\;\;(0.3T_{max}\;\leq t<0.7T_{max}\\ 0.1\;\;\;\;\;\;\;\;\;\;\;\;\;(t\geq 0.7T_{\max}) \end{array}\right.)$$

To prevent the particle velocity from becoming uncontrolled, this paper introduces a velocity clipping mechanism in the velocity update formula. This mechanism ensures that the magnitude of each parameter update does not exceed 20% of its range, thereby preventing excessive adjustments that could lead to parameter out-of-bounds conditions or oscillations and enhancing the algorithm’s stability. The formula is shown in Equation (14).

(14) $$v_{i,d\;}(t+1)=clip(v_{i,d}(t+1),-v_{\max,d},v_{\max,d})\;$$

where $v_{\max,d}=0.2\cdot (x_{\max,d}-x_{\min,d})\;$, and $x_{\max,d}$ and $x_{\min,d}$ are the upper and lower bounds of the d-th dimensional parameter.

Finally, the trajectory similarity penalty term $\Delta v_{i}$ is incorporated into the velocity update formula of STPSO-BP, forming a multi-objective-driven hybrid optimization mechanism, as shown in Equation (15).

(15) $$v_{i\;}(t+1)=w\cdot v_{i}\;(t)+c_{1}r_{1}\;(pbest_{i}-x_{i}(t))+c_{2}r_{2}(gbest-x_{i}(t))+\alpha \cdot v_{LSTM\;}+\Delta v_{i}$$

### 4.4. Generation of Model Training Set Data and Parameter Settings

Through LHS, 3000 points are randomly selected from 10,000 reachable points during the manipulator’s operation to obtain its spatial poses. Using the MATLAB Robotic Toolbox for forward kinematics solutions, simulation data of six joint angles and corresponding end positions are obtained. Several groups are extracted and substituted into the forward kinematics model in Section 3 for verification. The results match the end positions, indicating the correctness of the manipulator model built in MATLAB. To avoid data correlation, the samples are randomly divided into three parts: 70% for the BP neural network training set, 15% for the validation set, and 15% for the test set. After determining the experimental data, a BP neural network program is written in MATLAB for training, with parameters continuously adjusted during the training process. After extensive training and comparing simulation results, the BP neural network parameters shown in Table 4 are finally selected.

Building on the construction logic of the BP training set, we further generate training data for the LSTM model. During PSO-based BP parameter optimization, particles’ historical optimal positions (personal best) across iterations for each training sample are recorded to form historical optimal matrices. These matrix sequences serve as LSTM inputs—optimal particle trajectories with explicit temporal features in parameter space. Finally, the actual optimal velocities between consecutive time steps are derived using Equation (16).

(16) $$v_{t}=p_{t+1}-p_{t}$$

Here, $p_{t}$ denotes the historical optimal position vector of the particle at iteration t, and $v_{t}$ the inter-step actual optimal velocity vector. $v_{t}$ reflects the particle movement trend during optimization, guiding the LSTM to learn trajectory–velocity mappings. Leveraging 3000 BP training tasks, 3000 complete LSTM training datasets are generated. Each dataset contains multi-particle trajectory and velocity sequences, forming high-quality time series for predictive LSTM modeling.

### 4.5. 6-DOF PD Detection Mechanical Arm End-Positioning Error Parameter

To evaluate the superiority of the IK model proposed in this paper over traditional methods, the end-positioning error parameter Δ*L* of the robotic arm is introduced as a quantitative assessment criterion [24]. In PD detection scenarios, varying detection methods and object structures necessitate different positioning accuracy requirements. The simulation focuses on a KYN28A-12 switchgear with a 210 mm × 150 mm observation window and 650 mm × 30 mm cabinet gaps. For the detection of the observation window, the geometric center of the window is set as the target detection point. The allowable range for positioning error is defined as a circular area with a diameter equal to the width of the observation window, that is, ΔL≤75 mm. In the case of cabinet gap detection, the error is restricted within the width of the gaps, namely, ΔL≤15 mm. Based on the error range requirements of these two detection scenarios, the radius (15 mm) of the cabinet gap width is set as the positioning error tolerance range in this paper [25], aiming to adapt to different detection accuracy requirements.

**Figure 7:**
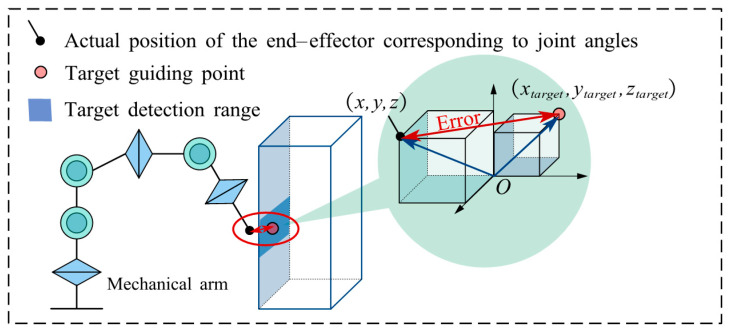
Schematic diagram for solving the end-effector positioning error parameter of the manipulators.

The detection process is shown in Figure 7. First, through the IK model, the position coordinates $\left(x_{target},\;y_{target},\;z_{target}\right)$ of the target detection point are mapped to the robotic arm joint angle vector $\theta =[\theta_{1},\theta_{2},\theta_{3},\theta_{4},\theta_{5},\theta_{6}]$. Then, based on the forward kinematics model, the actual position coordinates $\left(x_{actual},\;y_{actual},\;z_{actual}\right)$ of the end-effector under these joint angles are calculated. Finally, the deviation ΔL between the exact position and the target detection point is quantified using the Euclidean distance formula, as shown in Equation (17).

(17) $$\Delta L=\sqrt{{\left(x-x_{target}\right)}^{2}+{\left(y-y_{target}\right)}^{2}+{\left(z-z_{target}\right)}^{2}}$$

### 4.6. Comparative Analysis of End-Effector Positioning Error Compensation Based on the STPSO-BP Algorithm

**Figure 8:**
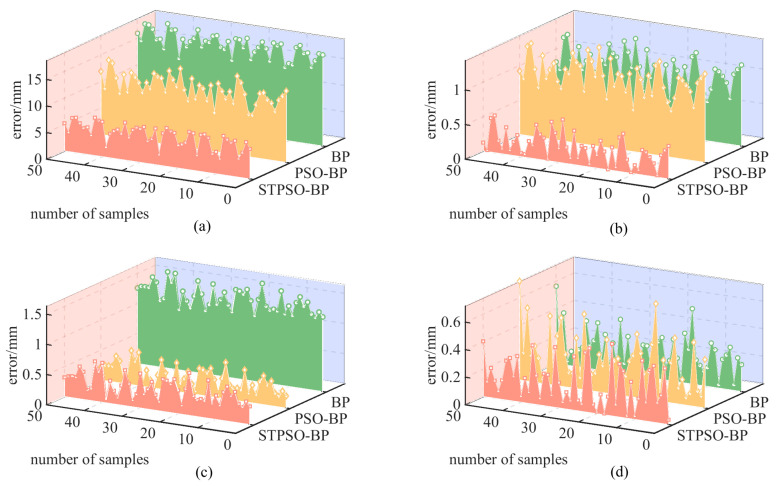
Comparison of end-effector positioning errors before and after improvement: (**a**) absolute positioning prediction errors across different algorithms; (**b**) prediction errors in the X-direction for various algorithms; (**c**) prediction errors in the Y-direction for distinct algorithms; (**d**) prediction errors in the Z-direction for different algorithms.

To validate STPSO-BP’s superiority in compensating robotic arm end-positioning errors, this study compares BP, PSO-BP, and STPSO-BP. Fifty points were randomly and uniformly sampled in the robot’s workspace, with errors derived as the mean of 10 repeated measurements. As visualized in Figure 8, the 50 sampled points reveal distinct error distribution patterns. The traditional BP algorithm exhibits large fluctuations and a broad error range. At the same time, PSO-BP and STPSO-BP show reduced variability.

Table 5 summarizes each algorithm’s average positioning error, calculated from 50 sampled points. The traditional BP algorithm produces a 17.48 mm error, failing to meet high-precision requirements. PSO-BP reduces the error to 10.78 mm via particle swarm optimization, verifying the effectiveness of parameter tuning. The improved STPSO-BP achieves a 5.43 mm error, a 68.9% and 49.6% reduction compared to BP and PSO-BP, respectively, demonstrating superior compensation capability. Axis-specific error analysis reveals the following: In the *X*-axis, STPSO-BP’s total error of 0.24 mm represents 76.6% and 73.3% reductions compared to PSO-BP (1.01 mm) and BP (0.90 mm). In the *Y*-axis, its 0.37 mm error is a 71.7% decrease from BP (1.31 mm), slightly higher than PSO-BP’s 0.28 mm. In the *Z*-axis, STPSO-BP’s 0.20 mm outperforms PSO-BP (0.28 mm) and BP (0.21 mm).

Compared with [26] (ISBOA-BP, 28.32 mm) and [7] (ASWO-BP, 32.00 mm), STPSO-BP reduces errors by 80.83% and 83.03%, respectively, highlighting significant precision gains. In summary, STPSO-BP efficiently optimizes model parameters, achieving optimal performance in end-positioning error compensation. As shown in Table 6, the STPSO-BP algorithm has an average computation time of 6.723046 ms, a standard deviation of 3.345526 ms, a maximum of 24.1601 ms, and a minimum of 3.9523 ms.

**Figure 9:**
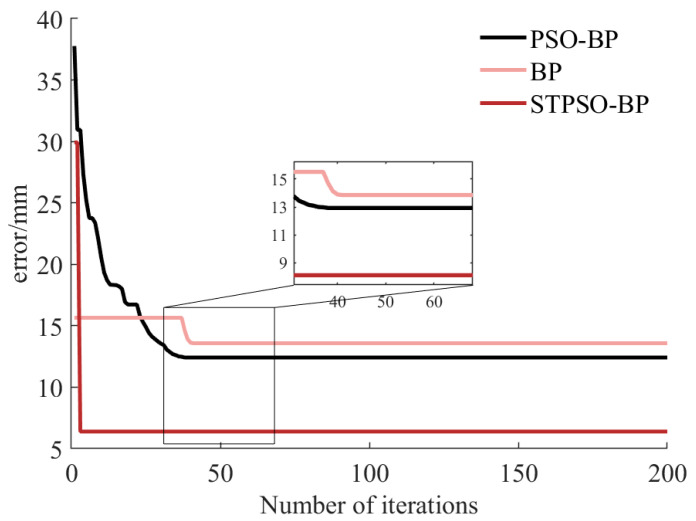
Error iteration comparison diagram of BP, PSO-BP, and STPSO-BP algorithms.

Figure 9 illustrates the evolution of minimum average errors during iterations of the BP, PSO-BP, and STPSO-BP algorithms. For convergence speed, the BP algorithm exhibits no initial error reduction and slow subsequent convergence, stabilizing after 40 iterations, highlighting its vulnerability to local optima. PSO-BP, leveraging PSO to optimize BP weights and biases, rapidly reduces errors between 20 and 40 iterations, outperforming BP. STPSO-BP, integrating LSTM temporal features and dynamic decay coefficients, achieves a sharp error drop within 0–20 iterations, exhibiting the fastest convergence.

In precision, the traditional BP stabilizes at 14–15 mm, PSO-BP reduces it to 12–13 mm, and STPSO-BP further reduces it to 6–7 mm. Trajectory similarity penalties and dynamic coefficients in STPSO-BP help maintain population diversity, thereby avoiding local optima. This enables STPSO-BP to outperform traditional algorithms comprehensively in terms of convergence speed, precision, and robustness. In summary, STPSO-BP demonstrates significant advantages in convergence efficiency and accuracy. It confirms that integrating temporal modeling with optimization strategies effectively enhances error compensation performance, making it suitable for high-precision tasks.

## 5. Establishment of an Energy Consumption Optimization Model for PD Detection Manipulators

In high-voltage switchgear PD detection, manipulator energy consumption is critical due to the large number of switchgears in substations [27]. Excessive energy consumption causes rapid battery depletion, requiring frequent charging and thus prolonging the detection cycle [28]. Therefore, energy optimization is crucial during the design and operation of manipulators. This paper develops an energy optimization model to minimize total energy consumption throughout the motion cycle. It performs energy efficiency optimization analysis and then plans the joint angle trajectory. In addition, we set the sum of the manipulator’s body weight and end load as m, with each joint’s dynamic parameters listed in Table 7.

When calculating the energy consumption of a six-joint robotic arm moving from its initial position to the end position, the dynamic characteristics of each joint, the end-motion characteristics, and the friction effect need to be comprehensively considered. First, the rotational kinetic energy of each joint is calculated. Subsequently, by summing up the rotational kinetic energy of all joints, the total rotational kinetic energy $E_{rot}$ of the robotic arm can be obtained, as shown in Equation (18).

(18) $$E_{rot}=\sum_{i=1}^{6}\frac{1}{2}I_{i}\omega_{i}^{2}$$

where $I_{i}$ is the moment of inertia of the *i*-th joint, $\omega_{i}$ is the average angular velocity of the *i*-th joint, and the specific values are shown in Table 7.

Subsequently, based on the robotic arm’s motion path, the change in gravitational potential energy $△E_{pot}$ is calculated. The change in gravitational potential energy is directly related to the change in the end-position height. The formula for calculating the change in gravitational potential energy is shown in Equation (19).

(19) $$△E_{pot}=mg(h_{final}-h_{initial})$$

where m is the total mass of the manipulator’s body and the end load, g is the acceleration due to gravity (9.81 m/s^2^), $h_{final}$ is the height of the end position at the final stage, and $h_{initial}$ is the height of the initial position.

During the end-effector’s motion, the translational kinetic energy $E_{\mathrm{trans}}$ is introduced due to the contribution of the linear velocity of the robotic arm’s end-effector. First, the average value of the joint angular velocity $\overset{\cdot }{q_{avg}}$ is mapped to the average linear velocity $v_{avg}$ and average angular velocity $\omega_{avg}$ of the end-effector through the geometric Jacobian matrix J(q) (where q is the joint angle vector). Finally, based on the average linear velocity $v_{avg}$ of the end-effector, the translational kinetic energy of the end-effector can be obtained. The calculation process is shown in Equations (20) and (21).

(20) $$\left[\begin{array}{c} \overset{-}{v}\\ \overset{-}{\omega } \end{array}\right]=J(q)\cdot \overset{\cdot }{q_{avg}}$$

(21) $$E_{\mathrm{trans}}=\frac{1}{2}mv_{avg}^{2}$$

In addition, friction loss $E_{fric}$ is a non-negligible energy consumption term during the movement of the manipulators. According to the total path length d that the manipulator’s end-effector passes from the starting point to the ending point, the friction loss is calculated. The total path length d is computed using discrete-point coordinates, as specified in Equation (22). The expression for friction loss is shown in Equation (23).

(22) $$d=\sum_{i=1}^{N-1}\left||p_{i+1}-p_{i}|\right|$$

(23) $$E_{fric}=k\cdot m\cdot d$$

where $p_{i}=(x_{i},y_{i},z_{i})$ are the coordinates of discrete points on the trajectory, ||⋅|| is the Euclidean distance, and k is the dynamic friction coefficient. The dynamic friction coefficient *k* is set to 0.05 in this work, based on the experimentally identified range [0.05,0.25] reported in Simoni et al. [29]. This value represents a conservative estimate reflecting systems with well-lubricated transmissions and low internal resistance. The selected value lies at the lower bound of realistic robotic joint friction values and is consistent with lightweight industrial manipulator characteristics.

Combining the above-mentioned energy components, the total energy consumption of the robotic arm is approximately the sum of the joint rotational kinetic energy, the end-effector translational kinetic energy, the change in gravitational potential energy, and the frictional losses. The calculation formula is shown in Equation (24). The objective of the energy consumption optimization model is to minimize the total energy consumption E during the robotic arm’s motion, as shown in Equation (25).

(24) $$E=E_{rot}+△E_{pot}+E_{trans}+E_{fric}$$

(25) $$\min E=\min\left(\sum_{i=1}^{6}\left(\frac{1}{2}I_{i}\omega_{i}^{2}\right)+mg(h_{final}-h_{initial})+\frac{1}{2}mv_{avg}^{2}+k\cdot m\cdot d\right)$$

Among them, *I_i_* represents the moment of inertia of the *i*-th joint, $\omega_{i}$ represents the average angular velocity of the *i*-th joint, *m* denotes the total mass of the manipulators and the end-load, *g* stands for the gravitational acceleration, *h_final_* and *h_initial_* represent the heights of the initial and final positions of the manipulators, *v_avg_* represents the average linear velocity at the end, *k* represents the dynamic friction coefficient, and *d* represents the total path length that the end-effector passes from the starting point to the ending point.

Specific boundary conditions constrain the structure and joint angles of the robot. These boundary conditions define the value range for each joint angle, delineated by a lower and an upper limit. In this way, the reachability of the robot’s pose can be determined [30]. Detailed parameter settings are shown in Table 2. The constraint conditions are expressed as inequalities, as shown in Equation (26), where $\theta_{i}$ (*i* = 1,2,…,6) are the six joint angles of the manipulators.

(26) $$s.t.\left\{\begin{array}{c} \begin{array}{c} -160{}^{\circ}\leq \theta_{1}\leq 160{}^{\circ}\\ -150{}^{\circ}\leq \theta_{2}\leq 15{}^{\circ}\\ -200{}^{\circ}\leq \theta_{3}\leq 80{}^{\circ} \end{array}\\ -180{}^{\circ}\leq \theta_{4}\leq 180{}^{\circ}\\ -120{}^{\circ}\leq \theta_{5}\leq 120{}^{\circ}\\ -180{}^{\circ}\leq \theta_{6}\leq 180{}^{\circ} \end{array}\right.$$

## 6. Dual-Layer Adaptive Optimization Model Based on End-Effector Positioning Error Compensation and Energy Consumption Optimization

In PD detection, manipulators’ precise positioning and energy-efficient operation are critical for efficiency and cost reduction. However, balancing positioning accuracy with energy optimization remains challenging [31]. This study employs STPSO-BP instead of traditional IK for high-precision positioning and an energy consumption optimization model for enhanced efficiency. Optimizing only for positioning errors or energy consumption is insufficient. A dual-layer adaptive model is built: the first layer compensates for end-positioning errors, and the second optimizes energy consumption. Chaotic mapping-based angular constraints, dynamic weights, and dual-objective temperature regulation achieve optimal balance.

### 6.1. Construction of Initial Solution Set

First, the STPSO-BP algorithm solves for six joint angles to minimize end-positioning error based on the target detection point and manipulator base coordinates. Next, random ±δ perturbations are applied to each joint angle from this solution to generate a six-joint angle range. Finally, this range constrains the initial solution set for energy-optimal motion planning. Specific constraints are in Equation (27).

(27) $$s.t.\left\{\begin{array}{c} \theta \prime =\underset{\theta }{\min}\left(Error(\theta )\right)\\ Threshold\_i=\;[\theta_{i}\prime -\delta \cdot |\theta_{i}\prime |,\;\theta_{i}\prime +\delta \;\cdot |\theta_{i}\prime |](i=1,2,\ldots ,6) \end{array}\right.$$

Among them, θ′ is the joint angle that minimizes Error(θ); $\theta_{i}\prime$ (i=1,2,…,6) is each element in θ′; Threshold_i is the angular range of the i joint angle; and δ is the perturbation ratio of the joint angle, with a value range of [0,1].

### 6.2. CM-SA Algorithm

The PD detection manipulator’s energy optimization model challenges conventional algorithms, involving complex linear and nonlinear constraints. Leveraging the simulated annealing algorithm’s constraint-handling capability [32], this study implements it in MATLAB with the following parameters: initial temperature of 100, decay rate of 0.95, and 1000 iterations per temperature. To balance positioning error and energy consumption optimization, this study introduces chaotic mapping-based angular constraints, dynamic weight adjustment, and dual-objective temperature regulation, thereby enhancing optimization efficiency [33].

Chaotic mapping is a dynamic mechanism based on nonlinear models. This paper employs the Logistic map for dynamic weight adjustment. The initial control parameters are generated by a random function, with subsequent updates performed through nonlinear iteration. Its sensitivity to initial values enables the generation of pseudo-random chaotic sequences [34]. This characteristic breaks the fixed pattern of weight adjustment, allowing the algorithm to balance error and energy consumption optimization flexibly. The Logistic map and weight calculation are shown in Equations (28) and (29).

(28) $$\left\{\begin{array}{l} r_{t+1}=4r_{t}(1-r_{t})(t\neq 0)\\ r_{t}=rand(t)(t=0) \end{array}\right.$$

(29) $$\left\{\begin{array}{l} \omega_{1}(t)=r_{t}\cdot \omega_{\min}+\frac{(\omega_{\max}-\omega_{\min})t}{T_{\max}}\\ \omega_{2}(t)=1-\omega_{1}(t) \end{array}\right.$$

where $r_{t}$ is the control parameter at time t, $r_{t+1}$ is the control parameter at time t+1, and $\omega_{\min}$ and $\omega_{\max}$ are the minimum and maximum values of the error weights, respectively.

In addition, chaotic mapping is also applied to the angular range constraint and the perturbation process. Based on the angular range constraint in Equation (27), the angular range is further perturbed using chaotic mapping, as shown in Equation (30), and the initial solution $x_{0}$ is generated based on this angular range. In the perturbation session of the solution, the perturbation increment is guided by the chaotic sequence to enhance the randomness of the new solution exploration and to avoid falling into the local optimum, as shown in Equation (31).

(30) $$Threshold\_i=\;[\theta_{i}\prime -\delta \cdot |\theta_{i}\prime |\cdot (2r_{t}-1),\;\theta_{i}\prime +\delta \;\cdot |\theta_{i}\prime |\cdot (2r_{t}-1)](i=1,2,\ldots ,6)$$

(31) $$x_{j}=x_{i}+\Delta x\cdot r_{t}$$

Subsequently, a bi-objective-driven temperature dynamic adjustment strategy is introduced into the simulated annealing algorithm to comprehensively evaluate the overall quality of the optimization results and adjust the optimization strategy in real-time. In this paper, the end-positioning error (E) and energy consumption (C) are applied to calculate the normalization index α, as shown in Equation (32). The normalization index α is the key basis for temperature adjustment. When α decreases continuously and is below the threshold, the temperature decay rate is set to 0.98. By slowing the rate of temperature decrease, it becomes possible to search finely in the more favorable region. When α fluctuates significantly, the temperature decay rate is set to 0.90. By speeding up the temperature decline rate, the algorithm converges faster to prevent falling into local optima.

(32) $$\alpha =\omega_{1}\cdot E+\omega_{2}\cdot C$$

In summary, the dual-layer adaptive optimization model’s core objective is to coordinate each layer’s optimization mechanisms. Through the random perturbation strategy, initial population constraints, and adaptive temperature regulation, the manipulators can reach the target detection position while minimizing energy consumption, ultimately realizing comprehensive optimization of the manipulator’s performance. The iterative process of the algorithm for solving this model plan is shown below.

### 6.3. Comparative Validation Analysis of End-Effector Positioning Error Compensation and Energy Consumption Optimization Based on Dual-Layer Adaptive Optimization Model

**Figure 10:**
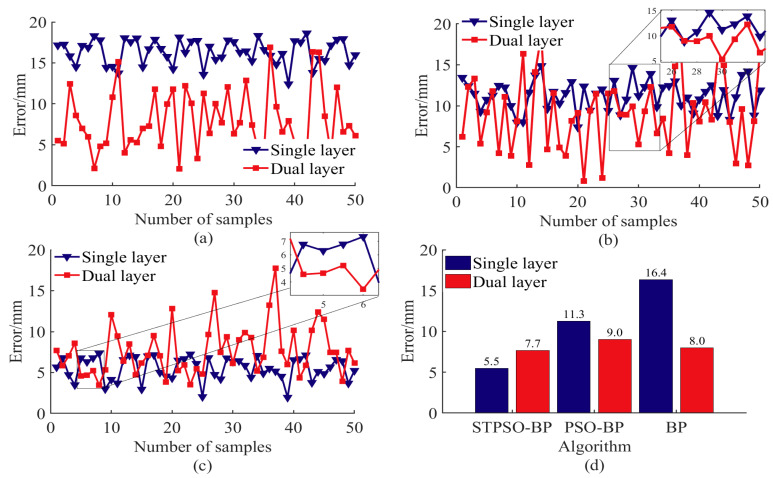
Comparative analysis of positioning errors between single-layer and dual-layer models under different error compensation algorithms: (**a**) BP neural network; (**b**) PSO-BP; (**c**) STPSO-BP; (**d**) comparison of mean positioning errors of single-layer and dual-layer models under different algorithms.

**Figure 11:**
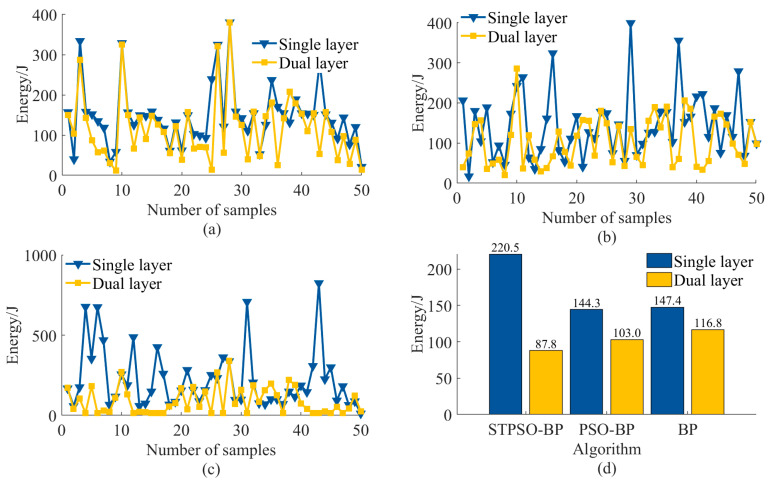
Comparative analysis of energy consumption between single-layer and dual-layer models under different error compensation algorithms: (**a**) BP neural network; (**b**) PSO-BP; (**c**) STPSO-BP; (**d**) comparison of mean energy consumption values of single-layer and dual-layer models under different algorithms.

The dual-layer adaptive optimization model for end-positioning error and energy consumption integrates energy optimization into the error compensation framework to achieve synergistic improvement. The single-layer model focuses solely on end-positioning error compensation, while the dual-layer model incorporates concurrent optimization of both error and energy consumption. For error compensation, the single-layer and dual-layer models employ traditional BP, PSO-BP, and STPSO-BP algorithms, respectively. Comparative experiments were conducted to evaluate model performance and algorithm impacts on error and energy metrics across frameworks. The manipulator was unloaded in the experiments, with its self-weight fixed at 35 kg. The analysis involved the random sampling of 50 points within the working range, with 10 repeated measurements taken at each point to calculate the mean values. Results are presented in Figure 10 and Figure 11.

In analyzing end-positioning error compensation, the three algorithms exhibit significant performance differences between single-layer and dual-layer models. As shown in Figure 10a,d, the traditional BP single-layer model has a mean error of 16.4 mm, with 78% of samples exceeding the 15 mm tolerance. Gradient descent optimization tends to converge to local optima, resulting in the highest errors but low volatility [35]. After dual-layer optimization, the mean error drops to 8.0 mm (a 50.22% reduction), with 8% of samples still out of tolerance. The increased volatility suggests that the CM-SA algorithm reduces errors by expanding the solution space at the cost of stability.

As shown in Figure 10b,d, the PSO-BP single-layer model achieves a mean error of 11.3 mm, with all samples meeting the tolerance and low volatility. This indicates that particle swarm optimization effectively reduces errors by expanding the search range [36]. After dual-layer optimization, the mean error decreases to 9.0 mm (a 20.35% reduction), but 10% of samples exceed the tolerance, and volatility increases. The limited improvement in the dual layer is constrained by the single layer’s low initial error and solution space. Thus, this results in lower accuracy compared to STPSO-BP.

As shown in Figure 10c,d, the STPSO-BP single-layer model performs optimally, with a mean error of 5.5 mm, full tolerance compliance, and minimal volatility. This suggests that STPSO’s global search has achieved near-optimal error minimization. During dual-layer optimization, the CM-SA algorithm balances error and energy consumption, increasing the mean error to 7.0 mm (a 21.43% increase) while maintaining full compliance. Thus, it trades a slight increase in error for a significant reduction in energy consumption.

The proposed dual-level adaptive optimization model reduces energy consumption via hierarchical collaboration. Level 1 (BP, PSO-BP, STPSO-BP) generates initial joint angles via IK, addressing end-effector positioning errors. Level 2 (CM-SA) uses initial angles with three mechanisms: (1) Chaotic mapping-based angular constraint minimizes torque fluctuations, cutting peak torque. (2) Dual-objective temperature regulation and Logistic map-based dynamic weight smooth trajectories, eliminating speed/acceleration abruptness and inertia losses. (3) Compressed solution spaces eliminate redundant paths, shortening motor runtime and cumulative energy use.

Traditional BP, relying on gradient descent, easily falls into local optima, causing high joint angle redundancy, often with unnecessarily large rotations or detours. As shown in Table 8, its average end trajectory reaches 2192.04 mm (the longest), extending motor runtime and task cycles, and increasing total energy. High redundancy forces CM-SA to prioritize correcting large deviations, inducing drastic angle changes, high torque peaks, and significant heat loss. Though CM-SA adjusts via chaotic weights, residual redundant trajectories remain due to high initial redundancy, limiting energy optimization. As shown in Figure 11a,d, single-layer energy averages 147.4 J, dropping to 116.8 J after optimization—a mere 20.76% reduction. BP’s low-quality initial solutions restrict CM-SA’s potential; resources focus on correction rather than refined energy regulation.

PSO-BP adjusts BP’s weights via PSO, avoiding some local optima, with lower initial joint redundancy than BP. However, PSO’s premature convergence leaves residual non-optimal angles, keeping redundant trajectories high. As shown in Table 8, its average end trajectory is 1887.14 mm (medium), indicating highly redundant trajectories. CM-SA reduces redundant segments via chaotic mapping but cannot eliminate them due to residual local optima. These redundant trajectories cause large speed fluctuations and frequent acceleration switches, increasing losses. Regarding, PSO-BP (Figure 11b,d), for 50 targets, the single-layer average energy is 144.3 J and is 103.0 J post-optimization (28.62% drop). The energy standard deviation is 12.7 J (30.6% lower than BP). Thus, its energy reduction outperforms BP but is slightly worse than STPSO-BP’s two-level model.

STPSO-BP (Figure 11a,d) achieves the most significant energy reduction for 50 targets: single-layer average energy is 220.5 J, dropping to 87.8 J after optimization (a 60.18% decrease), with highly concentrated energy distribution and low volatility. This superiority, even with the same CM-SA optimizer, stems from three key synergies:

(1) Spatio-temporal particle memory combined with LSTM predicts velocity direction, reducing random exploration and invalid joint rotation redundancy. (2) The trajectory similarity penalty maintains diversity via a repulsive force when similarity exceeds a threshold, avoiding repetitive sub-optimal angles. (3) STPSO optimizes BP weights and biases, improving IK fitting accuracy and reducing the redundancy of additional rotations for error compensation. (4) CM-SA performs refined energy optimization on STPSO-BP’s low-redundancy initial joint angles without excessive angle corrections, leading to a 60.18% energy reduction (Figure 11d) and the shortest average end trajectory (1699.64 mm, Table 8).

**Figure 12:**
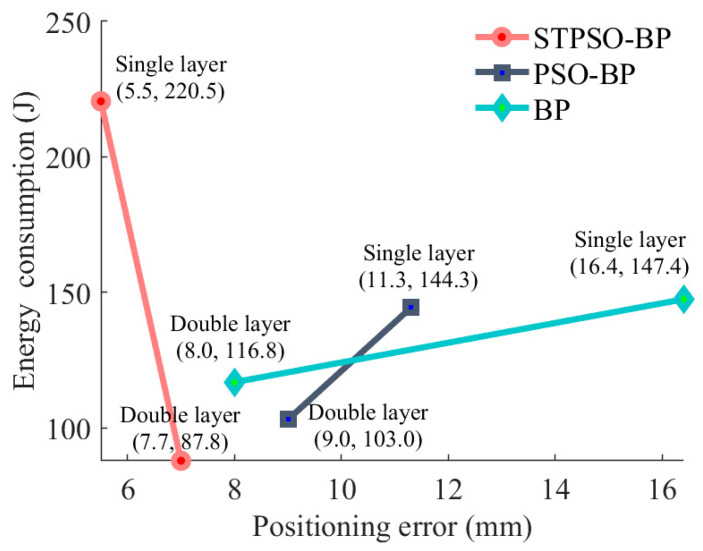
Plot of the relationship between positioning error and energy consumption for different models under single-layer and dual-layer structures.

Figure 12 shows the STPSO-BP dual-level model’s 7.70 mm end-positioning error, which is 3.75% lower than the traditional BP dual-level model (8.0 mm), and 14.44% lower than the PSO-BP dual-level model (9.0 mm). Compared with [26] (ISBOA-BP, 28.32 mm) and [7] (ASWO-BP, 32.00 mm), errors drop by 72.81% and 75.94%, well below the 15 mm precision threshold.

In energy optimization, STPSO-BP integrated with CM-SA reduces energy from 220.5 J (single-layer model) to 87.8 J, a 60.18% decrease. In contrast, the BP dual-level model only achieves a 25.0% drop, and the PSO-BP dual-level model a 14.76% reduction. Compared with [9] (SQP, 13.95% reduction), [8] (DRL, 23.21% reduction), and [10] (IHHO, 18.59% increase), the proposed method realizes larger energy savings while stabilizing the error at 7.70 mm, demonstrating precision–energy synergy.

As shown in Table 9, the STPSO-BP dual-level model’s average computation time is 51.60603 ms (SD: 68.41635 ms, max: 489.516 ms, min: 25.4219 ms). Although longer than the standalone STPSO-BP, this arises from its complex structure, increased variables, and expanded search space. Nevertheless, it remains within the acceptable online latency for PD detection manipulators, verifying practicality.

### 6.4. The Universality and Overall Control Flow of PD Detection Manipulators

For PD detection of high-voltage switchgears, end-position error compensation, energy optimization, and broad applicability of the dual-layer model are crucial for the robotic arm. Traditional analytical methods require deriving IK equations for robotic arm structures; configuration changes necessitate remodeling, limiting generality.

The STPSO-BP algorithm solves IK by learning pose–joint angle mapping rules across different D-H parameter systems via data. Thus, when D-H parameters change, only the training dataset with new configurations of pose–joint angle correspondences needs updating (Section 4.4); adaptation is achievable without modifying the network structure. This BP neural network-based strategy aligns with recent research trends. For example, Diprasetya et al.’s [37] KineNN framework adapts via neural networks to multi-configuration data of six-joint UR5 and five-joint UR3. Limoyo [38] achieve cross-configuration adaptation for differently configured robotic arms by combining graph neural networks.

But unlike BP neural network solutions in the above literature, this study innovates with a dual-layer modular architecture. STPSO-BP handles IK, CM-SA manages energy optimization, and the two collaborate to optimize joint angle combinations. Firstly, the first-layer STPSO-BP enhances the BP neural network’s data learning capability by introducing PSO and LSTM. It effectively replaces IK for joint angle solution, minimizing positioning error. Secondly, the second-layer CM-SA dynamically adjusts joint angle disturbance ranges via chaotic sequences through chaotic mapping. This allows the model to adaptively fit the constraint boundaries of the newly configured robotic arm during optimization, eliminating the need for constraint reconstruction. It also enables CM-SA to focus on optimizing torque, velocity, and trajectory instead of correcting large errors, thus achieving energy savings.

**Figure 13:**
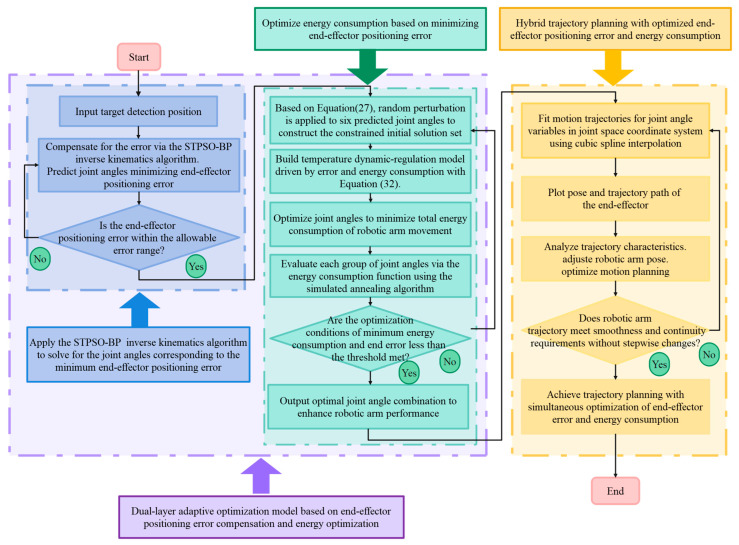
Algorithm control flow diagram for PD detection manipulators.

In terms of computation time, the STPSO-BP algorithm averages 6.7 ms (Table 6) for retraining, and the dual-layer model averages 51.6 ms (Table 9) for computation. Thus, the model’s lightweight migration cost meets real-time needs, further confirming its generality. The multi-model fusion process is shown in Figure 13.

## 7. Simulation Experimental Verification

### 7.1. Initial Attitude and Position of Manipulators

**Figure 14:**
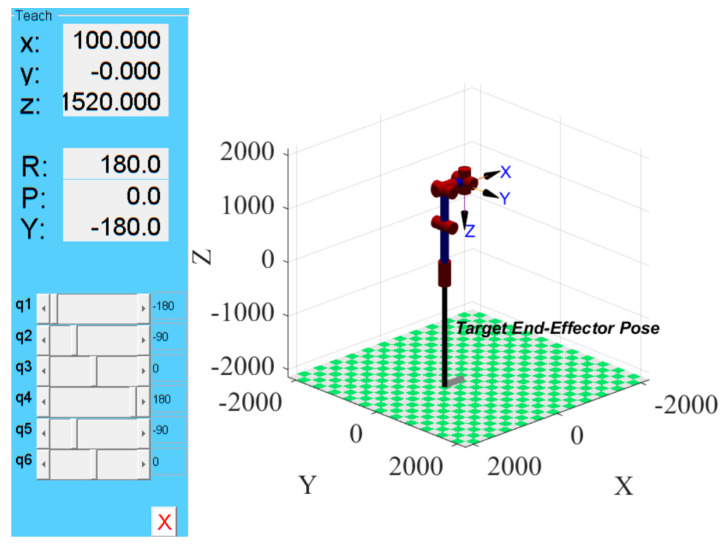
Initial pose of the PD detection manipulators.

**Figure 15:**
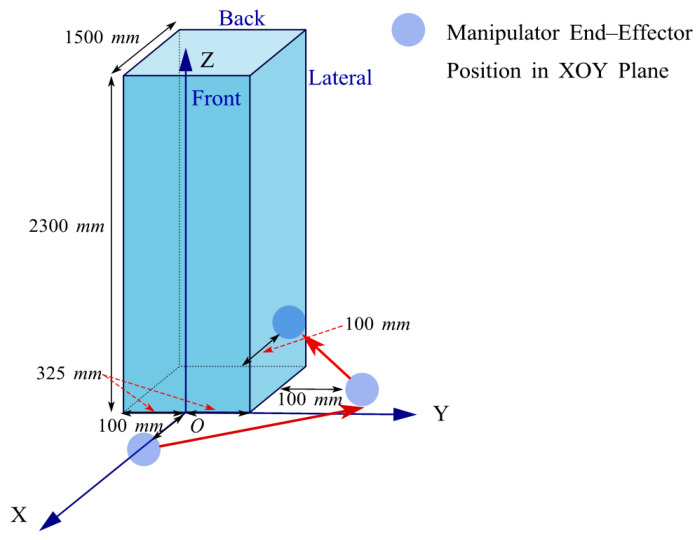
Establishing the initial position of the PD detection robot.

The simulation experiments were conducted on a 64-bit Windows 11 system with 32 GB of RAM, using MATLAB 2022b as the experimental platform. Based on the D-H parameters shown in Table 1 and the joint rotation ranges listed in Table 2, a 6-DOF manipulator model for PD detection was constructed using the Robotics Toolbox. By setting the initial joint states of the PD detection manipulator as *θ*_1_= 0°, *θ*_2_= −90°, *θ*_3_= 0°, *θ*_4_= 180°, *θ*_5_= −90°, and *θ*_6_= 0°, and the base coordinates as (0, 0, 0), we obtained the initial pose shown in Figure 14. For the KYN28A-12 high-voltage switchgear (650 × 1500 × 2300 mm), a coordinate system was defined with origin O at the front midpoint of the cabinet, for axes along the width (*Y*-axis), the depth (*X*-axis), and the height (*Z*-axis). By constraining the end-effector projection to maintain a 100 mm vertical distance from each surface, the end positions on the front, side, and rear planes were determined. Subsequently, the initial projection positions of the end-effector are obtained, as shown in Figure 15. Finally, the drive module follows the red path in the figure to ensure a constant 100 mm distance between the end projection and each surface in the initial pose.

To verify the feasibility of using the PD detection manipulator for PD detection in high-voltage switchgear, comparative experiments were conducted using three PD detection methods: AE, TEV, and UHF. We configured corresponding instruments and equipment, with the instrument names, models, and key technical parameters of the three methods listed in Table 10. The manipulator enables multi-dimensional and multi-physical signal detection of the switchgear by employing various instruments. Due to the different instruments held at the manipulator’s end under different detection methods, the total mass of the manipulator varies. The manipulator’s unloaded weight is 35 kg, and end-load instrument masses for various detection methods are shown in Table 10.

### 7.2. Global Detection Scheme for Manipulators with Different PD Detection Methods

#### 7.2.1. Coordinates of Target Detection Points Under Different PD Detection Methods

Taking the midpoint of the front side of the high-voltage switchgear as the coordinate origin, the high-voltage switchgear is modeled according to the IEC 60270 standard [39]. The distribution of UHF, AE, and TEV target points is shown in Table 11. With the manipulator as the origin, the switchgear detection range is *x* ∈ [0, 100] mm, y ∈ [−325, 325] mm, z ∈ [354, 1878] mm. As stated in Section 4.5, the manipulator’s working range is x ∈ [−1107, 1167] mm, y ∈ [−1169, 1175] mm, z  ∈ [213, 2070] mm. All target points lie within its working range. This indicates that moderately extending the base link can meet the demand for a large vertical span in the switchgear detection area, demonstrating the feasibility of the established manipulator model.

#### 7.2.2. Planning Scheme of Manipulator End Detection Path and Base Displacement Path Under Different PD Detection Methods

**Figure 16:**
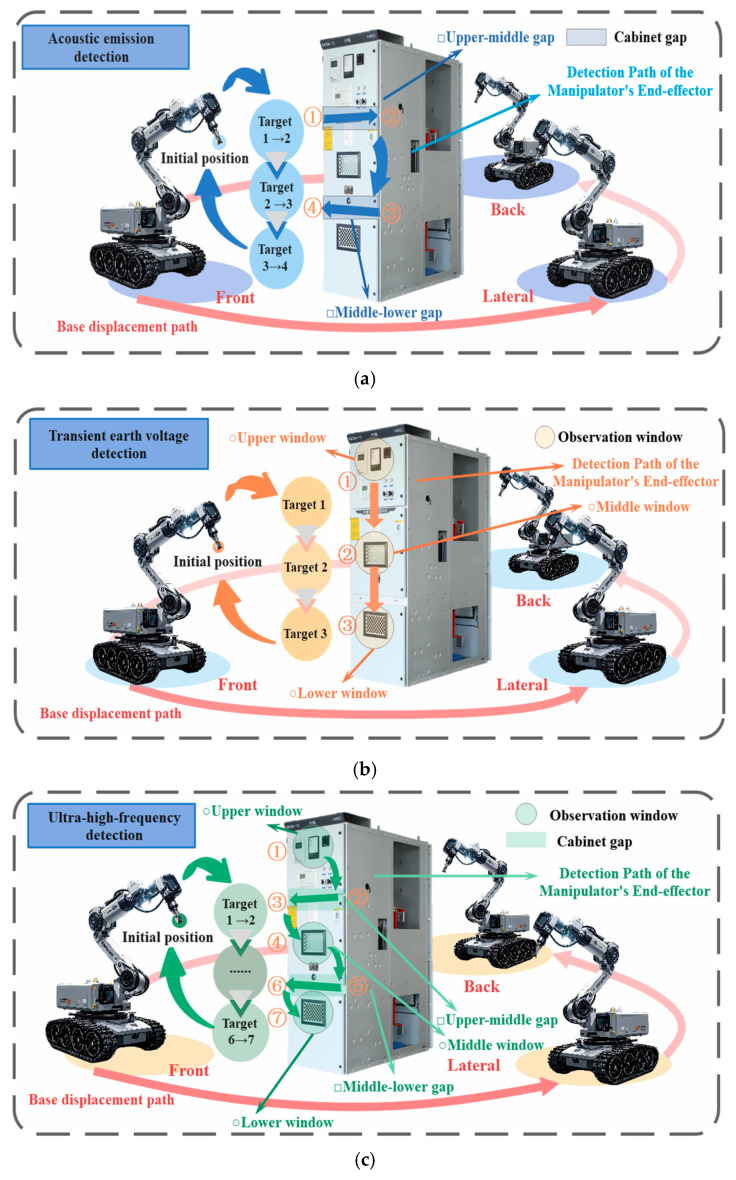
Manipulator detection trajectory and base displacement path: (**a**) AE detection; (**b**) TEV detection; (**c**) UHF detection.

Based on the coordinates of the target points, the detection path for the manipulator end and the displacement path for the base are planned, as shown in Figure 16.

Figure 16a presents the AE detection path. The manipulator end-effector starts from the default initial position. It plans a trajectory to Target 1 at the starting end of the upper-middle cabinet gap on the detection surface. After detection, it plans a path to Target 2 based on the gap’s endpoints. This process repeats until Target 4 is detected, when the manipulator resets. Then, the base moves to the next detection surface along a preset path. Figure 16b shows the TEV detection path planning. With the end-effector’s initial pose unchanged, spatial trajectory planning targets the upper observation window as Target 1. It then moves sequentially to targets, such as the middle observation window. Single-surface detection ends after Target 3 is inspected, and the manipulator resets before the base proceeds to the next surface. Figure 16c corresponds to UHF detection. Starting from the default position, the manipulator plans a trajectory to the upper observation window (Target 1). After completion, the end-effector moves to the starting end of the upper-middle cabinet gap (Target 2). Then, it follows it to the end (Target 3); after iteratively detecting up to Target 7, the manipulator resets. Finally, the base travels to the next detection surface’s starting point.

### 7.3. Analysis of Optimization Results Under Different PD Detection Methods

#### 7.3.1. Compensation for End-Positioning Errors and Energy Consumption Optimization Results

This paper completes path planning for end-effector detection and base displacement under different detection methods. Subsequently, PD detection manipulators’ end-positioning errors and energy consumption were compared for AE, TEV, and UHF methods. The end-positioning error and energy consumption data for practical detection scenarios are presented in Table 12. The horizontal dimension of Table 12 comprises three detection directions: forward, lateral, and backward. The vertical dimension encompasses errors at measurement locations, such as the upper observation window and the upper-middle compartment gap, as well as key parameters, including directional average errors, overall average error, and total energy consumption.

Table 12 shows that end-positioning errors for all three methods are effectively controlled within 3.58–11.34 mm. Thus, all errors strictly meet the 15 mm tolerance range specified in Section 4.4. This validates the STPSO-BP algorithm’s effectiveness for error compensation and the scheme’s adaptability to different PD detection methods. In accuracy, TEV detection has a total average error of 7.21 mm, with AE and UHF errors 5.69% and 10.68% higher, respectively. AE has 1361.38 J of energy consumption, while TEV and UHF consume 57.09% and 101.16% more, respectively. Despite energy differences, all values are reasonable for completing the corresponding detection tasks.

To verify the two-layer adaptive model’s robustness across scenarios, we analyze localization errors of 50 random samples (Section 6.3) and 30 actual detection points. First, Shapiro–Wilk tests confirm neither dataset follows a normal distribution; thus, independent Mann–Whitney tests are used.

**Figure 17:**
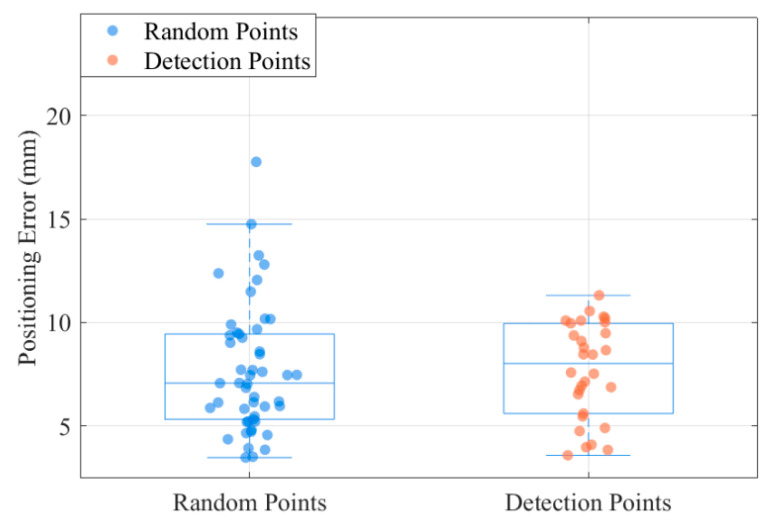
The data distribution of positioning errors between random points and detection points.

Analysis of the boxplot in Figure 17 reveals highly consistent error distributions between random and detection points. The random samples exhibit a marginally lower median (approximately 7 mm) than the detection points (approximately 8 mm), with minimal discrepancy. Their interquartile ranges overlap significantly, indicating similar distribution ranges and central tendencies. Random samples show a wider error range (some >15 mm with outliers); detection points cluster at 5–10 mm, which is more stable. Overall, the visualization supports STPSO-BP’s consistent localization accuracy in simulations and real tests.

As shown in Table 13, the Mann–Whitney test yields ***p*** = 0.564 (>0.05), indicating no statistical difference. Effect size Cohen’s d = 0.002 (<0.2), meaning there is a negligible difference in magnitude. The descriptive statistics for the datasets are as follows: mean, 7.70 mm and 7.64 mm; and standard deviation, 3.078 mm and 3.253 mm. Similar means and standard deviations indicate comparable dispersion between the two datasets.

In summary, STPSO-BP shows no significant error differences across scenarios, with minimal effect size and similar standard deviations, verifying its stability and robustness for manipulator localization in simulations and real tests.

**Figure 18:**
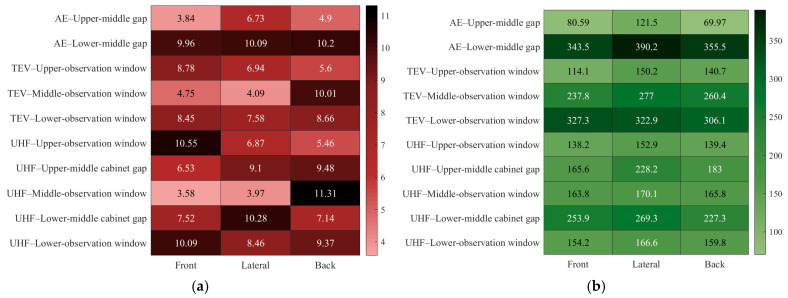
Heatmaps of error and energy consumption distribution for each detection surface under different detection methods: (**a**) heatmap of error distribution; (**b**) heatmap of energy consumption distribution.

**Figure 19:**
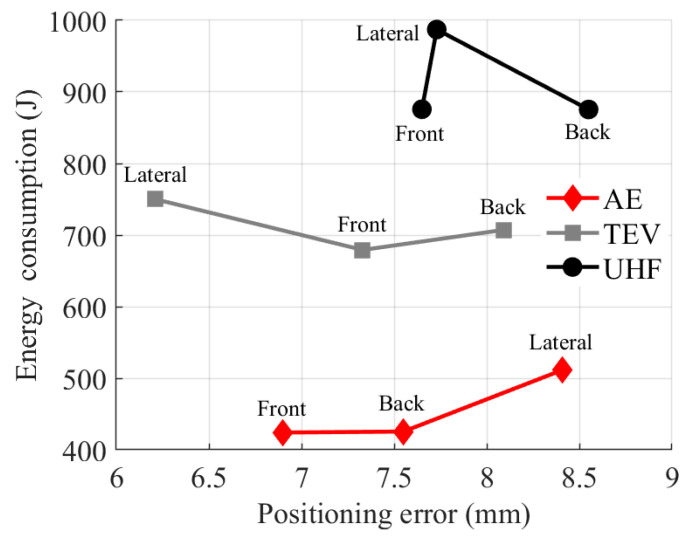
Relationship between average positioning error and total energy consumption for each detection surface under different detection methods.

Visualization analysis of the Table 12 data yielded error/energy consumption heatmaps and a positioning error–energy consumption line chart, as shown in Figure 18 and Figure 19. These heatmaps utilize color gradients to intuitively represent magnitudes for quick identification across detection points and directions.

Figure 18a shows the error distribution heatmap. AE, UHF, and TEV detections have significant color gradient differences in errors across switchgear components and directions. This corresponds to the more significant fluctuations in end-positioning errors of the 50 random sample points after optimization by the dual-layer adaptive optimization model in Section 6.3. After optimization, all positioning errors are within 15 mm. This fully demonstrates the effectiveness and reliability of the proposed dual-layer adaptive optimization model in error control.

Figure 18b shows the consumption distribution heatmap. The color gradient differences in energy consumption among the three detections are minor compared to those in errors. Energy consumption at the duplicate detection point’s front, side, and rear positions remains comparable. However, lateral directions show more significant differences between the front and rear. Notably, energy consumption was correlated with manipulator position, target location, and detection method complexity.

In summary, the spatial distribution variability of detection points across methods induces diversity in inverse kinematic solutions and heterogeneity in trajectory planning. These factors, combined with kinematic chain error transmission and multi-objective optimization conflicts, further complicate the situation. This results in heterogeneous distributions of manipulator positioning errors and energy consumption.

As shown in Figure 19, positioning error–energy consumption line charts compare performance across detection methods. The analysis reveals that lateral total energy consumption is significantly higher than the front and rear for all methods. The high-voltage switchgear’s depth of 1500 mm exceeds its width of 650 mm, causing longer lateral horizontal displacement and larger joint movements, thus increasing energy use and validating the energy model’s geometric sensitivity.

Considering the detection methods, AE detection has a total energy consumption of 1361.28 J, the lowest of all. Its 87.53 J energy span across directions is the largest, showing significant detection direction influence. It has an average end-positioning error of 7.62 mm, slightly higher than that of TEV, and a cross-directional error span of only 1.51 mm. It offers a balanced performance suitable for energy-sensitive long-term monitoring tasks with moderate error tolerance. TEV detection has a total energy consumption of 2136.47 J, the second lowest, with an energy span of 70.86 J, the smallest, showing highly controllable energy distribution. Its average end error is 7.21 mm, the smallest, but the cross-directional error span is 1.88 mm, the largest. With high overall precision, it is suitable for critical scenarios requiring strict accuracy. UHF detection has the highest total energy consumption, at 2738.34 J, and the most significant average error, at 7.98 mm. However, its cross-directional error span is 0.9 mm, the smallest, reflecting the most uniform error distribution, while its energy span is 111.72 J, the largest. It is suitable for scenarios that require complex discharge signal analysis with high tolerance for energy consumption. This analysis provides data support for balancing precision and energy consumption in multi-objective optimization.

#### 7.3.2. Hybrid Trajectory Planning for Manipulators with Different Detection Methods

Trajectory planning is a fundamental task in robot control. Reasonable planning can enhance manipulator control precision, stability, and efficiency. With the enrichment of application scenarios, trajectory optimization objectives increase according to task types [40]. Therefore, this paper develops hybrid trajectory planning based on joint angle combinations that optimize end error and energy consumption.

**Figure 20:**
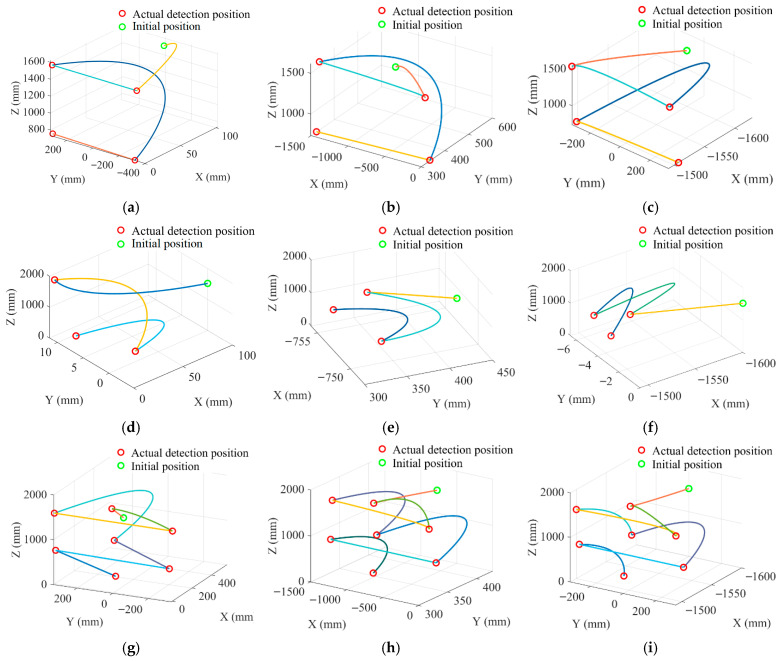
Trajectories of the manipulators for detecting different surfaces of high-voltage switchgear using TEV, UHF, and AE detection methods: (**a**) forward detection trajectory for AE; (**b**) lateral detection trajectory for AE; (**c**) rear detection trajectory for AE; (**d**) forward detection trajectory for TEV; (**e**) lateral detection trajectory for TEV; (**f**) rear detection trajectory for TEV; (**g**) forward detection trajectory for UHF; (**h**) lateral detection trajectory for UHF; (**i**) rear detection trajectory for UHF.

First, the dual-layer adaptive optimization model calculates joint angles and target positions. Then, cubic spline interpolation fits trajectories in the joint space to obtain motion paths for front, side, and rear detection target points, as shown in Figure 20. The circular arc trajectory consists of multiple spatial points, each corresponding to a unique end pose. Results show that end-effector trajectories under different detection methods are smooth and continuous without step changes, satisfying C^2^ continuity [41]. Figure 20 indicates that trajectory complexity correlates with energy consumption: UHF detection has the most complex trajectory and the highest total energy, while AE detection has the simplest trajectory and the lowest energy.

Additionally, comparing target positions with actual points in the trajectory shows deviations within a 15 mm tolerance. The dual-layer model achieves smooth trajectory control while ensuring optimal precision and energy consumption. This fully demonstrates its ability to accurately regulate manipulator motion trajectories, providing reliable support for practical tasks.

## 8. Discussion

### 8.1. Experimental Summary

First, comparative experiments were conducted. These aimed to analyze the positioning error compensation of the 6-DOF manipulator. Three approaches were compared: the STPSO-BP algorithm, the PSO-BP algorithm, and the traditional BP neural network. Their error compensation effects were evaluated, and experimental results were obtained. The STPSO-BP algorithm was found to exhibit significant advantages. This was especially true when processing high-dimensional error data. The error was minimized to 5.43 mm. Compared with the BP and PSO-BP algorithms, error reductions of 68.9% and 49.6%, respectively, were achieved.

Next, a comparative experiment analyzed the synergistic optimization of error and energy consumption between single-layer and dual-layer adaptive models. The dual-layer model demonstrated significant advantages, although optimization performance varied across different algorithms. Optimization for traditional BP neural networks reduced the average positioning error from 16.4 mm to 8.0 mm and energy consumption from 147.4 J to 116.8 J. However, increased error fluctuation caused some samples to exceed tolerance limits. In PSO-BP optimization, the average error decreased from 11.3 mm to 9.0 mm, but energy consumption dropped from 144.3 J to 103.0 J. However, out-of-tolerance samples rose from 20% to 40%. Compared to the above models, STPSO-BP optimization slightly increased the error from 5.5 mm to 7.0 mm but ensured full tolerance compliance with minimal fluctuation; meanwhile, energy consumption plummeted from 220.5 J to 87.8 J with converged fluctuations. This highlights an efficient balance between accuracy, stability, and energy consumption.

Furthermore, quantitative comparisons confirm superiority. Positioning accuracy: the STPSO-BP dual-layer model achieves a 7.70 mm error, >72% lower than ISBOA-BP [26] (28.32 mm) and ASWO-BP [7] (32.00 mm). Energy optimization: STPSO-BP combined with CM-SA reduces energy from 220.5 J (single-layer) to 87.8 J (60.18% drop). It outperforms SQP [9] (13.95% drop), DRL [8] (23.21% drop), and IHHO [10] (18.59% increase). It maintains 7.70 mm accuracy while realizing accuracy–energy synergy, showing advantages in complex solution spaces.

Finally, multi-objective detection experiments were conducted for TEV, UHF, and AE detection methods to systematically analyze their average errors, energy consumption, and end trajectories. Notably, all positioning errors ranged from 3.58 to 11.34 mm and fell within the 15 mm tolerance range, meeting the precision requirements for PD detection. Specifically, AE detection showed the lowest energy consumption at 1361.28 J. In comparison, TEV detection achieved the smallest error of 7.21 mm. Additionally, all trajectory planning results satisfied the C^2^ continuity requirement. These results fully validated the model’s adaptability to different detection scenarios.

### 8.2. Research Applicability

Regarding generality and cross-scenario adaptability, the dual-layer adaptive model is a data-driven grey-box model. It avoids complex analytical derivation relying on specific manipulator D-H parameters. By training with end pose–joint angle datasets for particular scenarios, it adapts to manipulators of varying configurations, DOFs, and sizes. This study uses a PD detection manipulator for high-voltage switchgear as a typical example, with an expandable algorithm framework.

Regarding DOF adaptability, the model yields more significant optimization gains for high-DOF manipulators. Their IK solution spaces are highly complex, with prominent accuracy–energy balance challenges. STPSO-BP breaks traditional analytical limits by learning nonlinear pose–joint angle mappings. CM-SA further optimizes energy using redundant DOF regulatory spaces. In contrast, low-DOF manipulators have limited solution spaces, weakening redundant regulation gains.

### 8.3. Research Limitations and Future Work

Despite strong performance, STPSO-BP-CM-SA incurs computational overhead and applicability limits. STPSO-BP, fusing PSO and BP, has an average inverse solution time of 6.72 ms. CM-SA in Layer 2 adds strategy perturbation, improving convergence but increasing time to 51.606 ms. Cycle-sensitive tasks require an adaptability assessment for this overhead. Regarding generality, the data-driven modular framework of the dual-layer model theoretically has potential for cross-configuration and cross-scenario adaptation. However, experimental validation has focused primarily on 6-DOF manipulators in high-voltage switchgear inspection tasks. Systematic validation across more diverse industrial scenarios and manipulator configurations with varying degrees of freedom remains pending.

Future work will expand the model’s validation scope to enhance generalization and practicality. We will test it under varying degrees of freedom, DH parameters, and detection scenarios to verify universal adaptability. Efforts to reduce task time and computational costs will improve engineering utility. Additionally, we will explore the model’s impact on motor dynamics via torque–current analysis and test robustness under extreme conditions to support industrial deployment.

## 9. Conclusions

This paper proposes a dual-layer adaptive optimization model that combines the STPSO-BP and CM-SA algorithms. Through end-positioning error comparisons, single- and dual-layer model contrasts, and multi-method simulations, the model’s effectiveness in enhancing manipulator performance is verified. Experimental results show that the dual-layer model reduces end-positioning error by 68.9% compared to the traditional BP algorithm. Meanwhile, energy consumption drops by 60.18% from the pre-optimization level. In conclusion, this model significantly improves the positioning accuracy of PD detection manipulators while cutting energy use. It offers an innovative approach for optimizing positioning errors and energy consumption in six-degree-of-freedom manipulators for this application.

## Figures and Tables

**Table 1:** Initial (D-H) parameters table for manipulators.

Joint *i*	*a_i_*/(cm)	*a_i_*/(°)	*d_i_*/(cm)
1	0	−90	89.5
2	65.8	0	0
3	3.5	−90	0
4	0	90	47.7
5	0	−90	0
6	0	0	6.8

**Table 2:** Rotation ranges of each joint.

Joint Angle	Angle Range	Joint Angle	Angle Range
$\theta_{1}$	[−160°, 160°]	$\theta_{4}$	[−180°, 180°]
$\theta_{2}$	[−150°, 15°]	$\theta_{5}$	[−120°, 120°]
$\theta_{3}$	[−200°, 80°]	$\theta_{6}$	[−180°, 180°]

**Table 3:** Key components of the manipulator’s electronic control system.

Joint	Motor	Encoder	Reducer	Driver	Motion Control Card
1	RE50	HEDS5540	GP Planetary Reducer + Worm and Worm Gear	Epos2 50/5	Turbo PMAC2- Eth-Lite Controller (Clipper)
2	RE50	HEDS5540		Epos2 50/5	
3	RE40	MR-L		ACJ-55-18	
4	EC-i52	HEDS5540	GP Planetary Reducer	ACJ-55-18	
5	EC-i40	HEDS5540		ACJ-55-18	
6	FHA-8C	Incremental Photoelectric Encoder	Harmonic Reducer	ACJ-55-18	

**Table 4:** BP network parameter values.

Maximum training epochs	10,000	Target performance	10^−13^
Learning rate	0.01	Maximum training time	6566

**Table 5:** Average end-effector positioning errors across algorithms.

Algorithm	Total Error	*X*-Axis	*Y*-Axis	*Z*-Axis
BP	17.48	0.90	1.31	0.21
PSO-BP	10.78	1.01	0.28	0.28
STPSO-BP	5.43	0.24	0.37	0.20

**Table 6:** Online calculation time.

	STPSO-BP Algorithm
Average calculation time (ms)	6.723046
Standard deviation (ms)	3.345526
Maximum calculation time (ms)	24.1601
Minimum calculation time (ms)	3.9523

**Table 7:** Rotation energy consumption parameters of joints.

Joint *i*	Moment of Inert (kg·m^2^)	Average Angular Velocity (rad/s)	Joint *i*	Moment of Inertia (kg·m^2^)	Average Angular Velocity (rad/s)
1	0.5	2.0	4	0.6	2.5
2	0.3	1.5	5	0.2	3.0
3	0.4	1.0	6	0.4	2.0

**Table 8:** Length of motion trajectory at the end of the robotic arm for various algorithms.

	BP Neural Network	PSO-BP	STPSO-BP
Average value (mm)	2192.04	1887.14	1699.64
Standard deviation (mm)	4.89	5.1380	5.10
Maximum value (mm)	2208.08	1910.00	1711.00
Minimum value (mm)	2188.23	1889.98	1690.40

**Table 9:** Length of motion trajectory at the end of the robotic arm.

	Dual-Layer Adaptive Optimization Model
Average calculation time (ms)	51.60603
Standard deviation (ms)	68.41635
Maximum calculation time (ms)	489.516
Minimum calculation time (ms)	25.4219

**Table 10:** Instrument configuration and technical parameters for three PD detection methods.

Detection Method	Instrument Name	Model	Main Technical Parameters	
AE Detection	Wideband Differential Sensor	WD	Operating frequency Range	125–1000 kHz
			Peak sensitivity (RefV/(m/s))	56 dB
			Dimensions	17.8 mm OD × 16.5 mm H
			Weight	20 g
TEV Detection	TEV Transient Earth Voltage Signal Sensor	iTEV	Detection bandwidth	3–100 MHz
			Measurement range	−40–60 dBmV
			Dimensions	60 mm × 60 mm × 35 mm
			Weight	100 g
UHF Detection	UHF Sensor	KPD2-UHF	Signal frequency band	300 MHz–1.5 GHz
			Product dimensions	Diameter 95 mm, thickness 40 mm
			Weight	0.46 kg

**Table 11:** Target detection points for high-voltage switchgear based on UHF, AE, and TEV detection.

		Front	Lateral	Back
AE	Upper-middle cabinet gap	(0, −325, 1562.08)	(0, 325, 1562.08)	(−1500, −325, 1562.08)
		(0, 325, 1562.08)	(−1500, 325, 1562.08)	(−1500, 325, 1562.08)
	Middle-lower cabinet gap	(0, −325, 737.92)	(0, 325, 737.92)	(−1500, −325, 737.92)
		(0, 325, 737.92)	(−1500, 325, 737.92)	(−1500, 325, 737.92)
UHF	Upper observation window	(0, 0, 1878.33)	(−750, 325, 1878.33)	(−1500, 0, 1878.33)
	Middle observation window	(0, 0, 1159.58)	(−750, 325, 1159.58)	(−1500, 0, 1159.58)
	Lower observation window	(0, 0, 354.58)	(−750, 325, 354.58)	(−1500, 0, 354.58)
TEV	Upper observation window	(0, 0, 1878.33)	(−750, 325, 1878.33)	(−1500, 0, 1878.33)
	Upper-middle cabinet gap	(0, −325, 1562.08)	(0, 325, 1562.08)	(−1500, 325, 1562.08)
		(0, 325, 1562.08)	(−1500, 325, 1562.08)	(−1500, −325, 1562.08)
	Middle observation window	(0, 0, 1159.58)	(−750, 325, 1159.58)	(−1500, 0, 1159.58)
	Middle-lower cabinet gap	(0, −325, 737.92)	(0, 325, 737.92)	(−1500, 325, 737.92)
		(0, 325, 737.92)	(−1500, 325, 737.92)	(−1500, −325, 737.92)
	Lower observation window	(0, 0, 354.58)	(−750, 325, 354.58)	(−1500, 0, 354.58)

**Table 12:** End-effector positioning error and energy consumption simulation results based on different detection methods.

	AE			TEV			UHF		
	Front	Lateral	Back	Front	Lateral	Back	Front	Lateral	Back
Upper observation window error /mm				8.78	6.94	5.60	10.55	6.87	5.46
Upper-middle cabinet gap error /mm	3.84	6.73	4.90				6.53	9.10	9.48
Middle observation window error /mm				4.75	4.09	10.01	3.58	3.97	11.31
Middle-lower cabinet gap error /mm	9.96	10.09	10.20				7.52	10.28	7.14
Lower observation window error /mm				8.45	7.58	8.66	10.09	8.46	9.37
Average error per direction /mm	6.90	8.41	7.55	7.33	6.21	8.09	7.65	7.73	8.55
Total average error /mm	7.62			7.21			7.98		
Upper observation window energy consumption /J				114.06	150.23	140.69	138.21	152.89	139.42
Upper-middle cabinet gap energy consumption /J	80.59	121.47	69.97				165.58	228.21	183.03
Middle observation window energy consumption /J				237.84	276.98	260.43	163.85	170.12	165.80
Middle-lower cabinet gap energy consumption /J	343.54	390.19	355.51				253.92	269.33	227.35
Lower observation window energy consumption /J				327.32	322.87	306.05	154.17	166.62	159.83
Total energy consumption per direction /J	424.13	511.66	425.48	679.22	750.08	707.17	875.73	987.17	875.45
Total energy consumption /J	1361.28			2136.47			2738.34		

**Table 13:** Results of the Mann–Whitney U test analysis.

Variable Name	Variable Value	Sample Size	Median	Standard Deviation	Mean	*p*	Median Difference	Cohen’s d
Positioning error	Random Points	50	7.069	3.078	7.70	0.564	0.946	0.002
	Detection Points	30	8.015	3.253	7.64			

## Data Availability

Key data supporting the findings of this study are included in this paper. A more complete dataset can be reasonably requested from the authors.
